# Sustained illness burden over time among Australians with myalgic encephalomyelitis/chronic fatigue syndrome

**DOI:** 10.1371/journal.pone.0338433

**Published:** 2025-12-29

**Authors:** Breanna Weigel, Natalie Eaton-Fitch, Kiran Thapaliya, Sonya Marshall-Gradisnik

**Affiliations:** 1 National Centre for Neuroimmunology and Emerging Diseases, Griffith University, Gold Coast, Queensland, Australia; 2 Consortium Health International for Myalgic Encephalomyelitis, Griffith University, Gold Coast, Queensland, Australia; 3 School of Pharmacy and Medical Sciences, Griffith University, Gold Coast, Queensland, Australia; University of Rijeka Faculty of Health Studies: Sveuciliste u Rijeci Fakultet zdravstvenih studija, CROATIA

## Abstract

**Background:**

Myalgic Encephalomyelitis/Chronic Fatigue Syndrome (ME/CFS) is a disabling chronic illness. Many people with ME/CFS (pwME/CFS) are unable to continue employment and require support to complete activities of daily living. Despite this, ME/CFS remains unrecognised as a disability in Australia. The present study aimed to highlight the profound burdens experienced by pwME/CFS over time to provide evidence of permanency and necessitate reforms to Australian healthcare policies.

**Methods:**

Data were collected for this longitudinal investigation between 1^st^ October 2021 and 3^rd^ October 2024. All participants were Australian residents aged between 18 and 65 years fulfilling the Canadian or International Consensus Criteria. Sociodemographic information, medical history, illness presentation and patient-reported outcomes were collected using three self-administered questionnaires distributed at approximately six-month intervals. Illness presentation and patient-reported outcomes were investigated over 12 months with Cochran’s *Q*, Friedman and one-way repeated measures ANOVA tests using Statistical Package for the Social Sciences version 29.0. Quality of life data were compared with Australian population norms using one-sample Wilcoxon signed-rank tests.

**Results:**

Thirty-two pwME/CFS (n = 22/32, 68.8% female) participated at all three time points. At baseline, the mean age was 44.03 years and median illness duration was 12.50 years. Participants reported a median of 30 symptoms at each time point — the most common of which were also the most severe in presentation. Importantly, there were no significant changes in any symptom or patient-reported outcome over the 12-month study period. Overall health status, physical health and the ability to participate in daily and work life activities were the most substantially impacted. Quality of life was significantly reduced among pwME/CFS when compared with population norms at all time points.

**Conclusions:**

PwME/CFS face substantial and sustained illness burdens. These consistent, profound impairments emphasise the need for improved access to disability and social support services for pwME/CFS in Australia through policy reform.

## Background

Myalgic Encephalomyelitis/Chronic Fatigue Syndrome (ME/CFS) is a complex, chronic illness associated with debilitating, multi-systemic symptoms [1–3]. Approximately 1% of the global population is affected by the illness [4]. ME/CFS is characterised by cognitive, autonomic and immune dysfunction, as well as disrupted sleep, myalgia, arthralgia and neurosensory disturbances [1–3,5–8]. Post-exertional malaise is the defining feature of ME/CFS and describes the worsening of symptoms following physical, mental or emotional exertion [1–3,5–8].

The spectrum of the illness presentation of ME/CFS ranges from a retained ability to participate in some premorbid life activities to being bed-bound and unable to complete basic self-care activities [2,3]. People with ME/CFS (pwME/CFS) may also fluctuate in their health status and experience periods of reduced impairment, as well as periods of severe and disabling illness [1–3,8]. As evidence-based treatment options are limited and less than 10% of pwME/CFS experience permanent remission, the prognosis of ME/CFS is almost always life-long illness [1–3,7–10].

Care protocols for ME/CFS typically involve a multidisciplinary team of healthcare professionals to manage symptoms and maximise functioning [1–3,8]. Many pwME/CFS require assistance to complete daily activities from support workers or carers (including informal carers, such as family or friends) or through the use of disability supports, such as mobility aids or home modifications [7,8,11,12].

There is also an extensive personal economic burden that is foisted upon pwME/CFS, as approximately 50% to 60% of pwME/CFS are unable to continue employment due to their illness [12–17]. Those with the capacity to continue employment are typically unable to work full-time [2,7,11,12,18,19]. In addition to lost income, Close *et al*. [19] reported that each Australian living with ME/CFS faces a direct, annual healthcare cost of approximately $20,000 according to patient-level data collected between 2017 and 2019. Hence, financial aid, care subsidies and access to federally funded disability support services, such as the National Disability Insurance Scheme, are imperative for pwME/CFS to not only optimise their health outcomes and quality of life (QoL), but also to prevent further deterioration in health [1–3,7,8,11].

However, pwME/CFS are frequently deemed ineligible and denied access to such necessary supports [11,20–23]. Despite previous Australian research documenting the extensive, disabling impacts of the illness [15,24,25], ME/CFS remains unrecognised as a disability in Australia [11,20]. The Australian Public Service Commission [26] defines disability as “a limitation, restriction or impairment, which has lasted, or is likely to last, for at least six months and restricts everyday activities.” However, fulfilment of this definition alone does not constitute eligibility to access essential disability support services, such as the Disability Support Pension and the National Disability Insurance Scheme [11,20]. The eligibility criteria for these services, instead, demand extensive supporting documentation, including biological evidence of illness to confirm the permanence of physically disabling conditions [11,20]. A biomarker that is both indicative of illness presence and proportional to illness severity or prognosis remains yet to be identified for ME/CFS and, consequently, is not a component of the condition’s current diagnostic criteria [1,2,4,7,9]. The most stringent diagnostic criteria for ME/CFS that are currently available, being the Canadian Consensus Criteria (CCC) [5] and International Consensus Criteria (ICC) [6], capture the illness duration and impact on daily activities requirements specified within the Australian Public Service Commission’s [26] definition of disability. Low remission rates further corroborate the largely permanent nature of ME/CFS [1–3,7–10]. Despite this, ME/CFS continues to be excluded from the list of acquired disabilities eligible for the National Disability Insurance Scheme — a program funded by the federal government designed to facilitate access to subsidised support services for people living with disability in Australia [11,20].

Support services may be accessed privately; however, this incurs extensive personal financial costs [7,18,19]. Multimorbidity and the use of several medications are also common among pwME/CFS and compound the financial burden of those affected [8,18,19,27]. This is further complicated by a lack of income support. Johnston *et al*. [16] and Eaton-Fitch *et al*. [15] reported that 42.2% and 49.3% of Australians with ME/CFS who were unemployed due to their illness, respectively, were not receiving the Disability Support Pension [15,16], which is Australia’s federally funded income support program.

It is imperative that the healthcare policies that govern access to disability and social support services in Australia be reformed to reflect the long-term, disabling symptoms experienced by pwME/CFS and fulfil the support needs of this illness cohort. The present study, therefore, aimed to highlight the profound and sustained burdens among pwME/CFS by documenting illness presentation and patient-reported outcomes over a 12-month period. Specifically, the present study served to answer the following research question: “Do the illness burdens (including symptom presentation and patient-reported outcomes) associated with ME/CFS change over time?”.

This is the first detailed, longitudinal analysis of the illness burdens faced by pwME/CFS in Australia. Such nation-specific data are integral to develop federal healthcare policies that are guided by the lived experiences of pwME/CFS. Patient-reported outcome measures (PROMs) employed in existing Australian research have been similarly used in this longitudinal study to foreground that the previously documented impairments in health and wellbeing among pwME/CFS are consistent and long-term.

## Methods

### Study design and participants

Data were collected for this longitudinal, prospective panel study at the National Centre for Neuroimmunology and Emerging Diseases (NCNED), Griffith University, Gold Coast campus, Queensland, Australia from 1^st^ October 2021–3^rd^ October 2024. The sampling frame consisted of approximately 1,500 people enrolled in the NCNED’s participant database, as previously described [15,28]. The database was screened for participants providing valid data within the last two months who were: a) aged between 18 and 65 years; b) residents of Australia; c) English speakers; d) non-smokers; and e) not currently pregnant or breastfeeding. Participants fulfilling this preliminary eligibility criteria (n = 246) were contacted with recruitment information.

The sociodemographic information, medical history and symptoms of those who responded to the recruitment invitations (n = 111) were collected to confirm their illness status, as well as to identify any exclusionary health conditions reported by the participants. This study required participants to both: a) report having received a formal diagnosis of ME/CFS from a physician and b) experience an illness presentation consistent with the CCC [5] or ICC [6]. Respondents reporting additional diagnoses that may explain or confound their symptoms or functional limitations were excluded. Exclusionary diagnoses included: a) genetic or metabolic diseases; b) active or previous (if in remission) autoimmune diseases; c) malignancy within the last five years; and d) premorbid history of mental health illness. Participants who reported non-exclusionary conditions (such as hypertension or polycystic ovary syndrome) but met all other eligibility criteria were retained in the study providing the additional diagnoses were believed to be controlled and were not substantially contributing to their symptoms.

**Fig 1:**
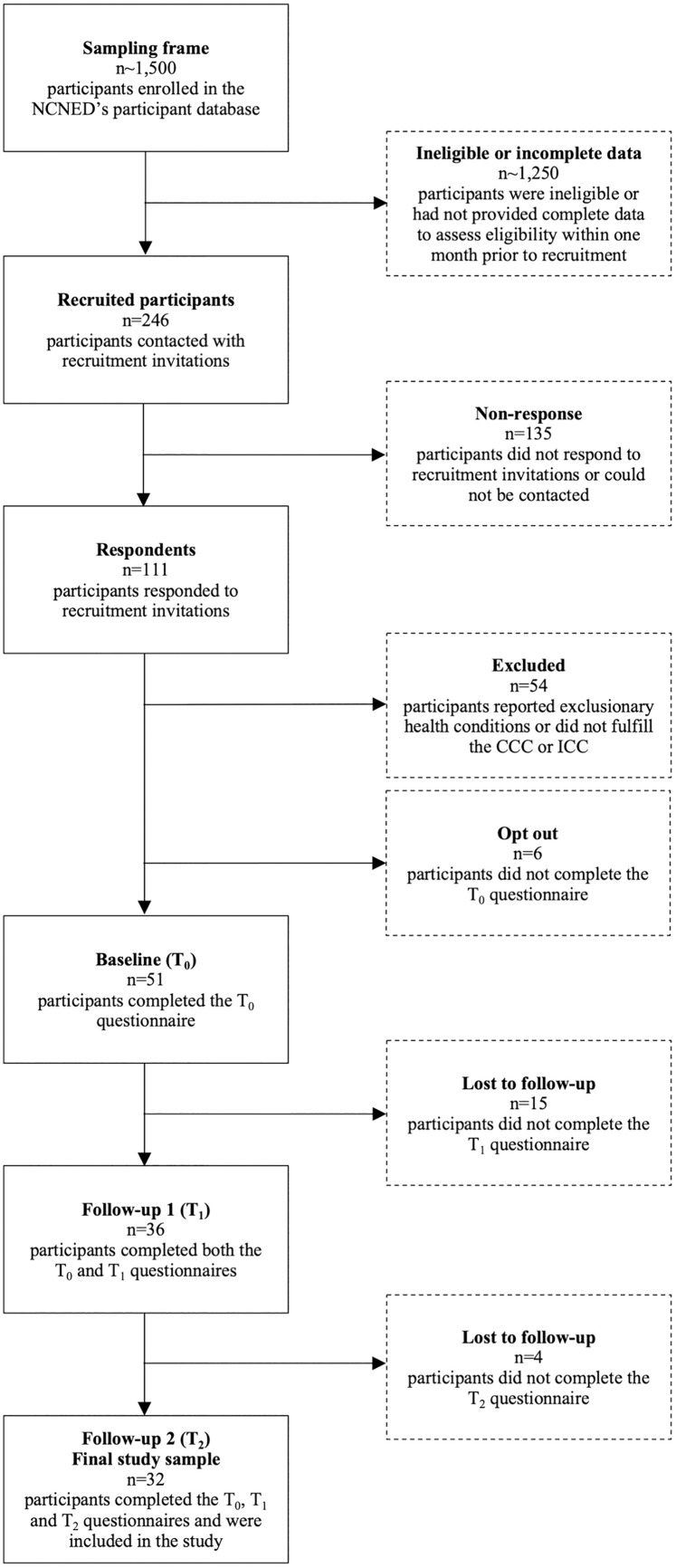
Participant recruitment, assessment for eligibility and study completion. Abbreviations: *CCC* Canadian Consensus Criteria; *ICC* International Consensus Criteria; *NCNED* National Centre for Neuroimmunology and Emerging Diseases.

Exclusion criteria were met by n = 54 respondents. An additional n = 6 participants opted out of the study and n = 19 participants did not complete the two follow-up questionnaires. The total number of eligible participants with valid data was, therefore, n = 32. Fig 1 depicts the exclusion and non-participation of participants at each stage of the present study.

Ethical approval was acquired for this study from the Griffith University Human Research Ethics Committee (Reference Number: 2021/518). Informed consent was obtained from all respondents during screening and at each time point via the completion of a mandatory consent question to grant access to the online questionnaires. Consent to the future use of collected data was also requested during screening in the event of a respondent’s non-participation. Unidentifiable group statistics of the non-participants (including participants lost to follow-up and ineligible respondents) are only reported herein for those who consented to the future use of their data (n = 19 and n = 18, respectively). All n = 32 pwME/CFS who participated at all three time points of the present study provided their informed consent to participate in the research and to publish unidentifiable group results prior to commencing the questionnaire at each time point.

The study design and methods of data collection and storage have been informed by and comply with the Griffith University Ethics Manual [29], the Australian Government National Health and Medical Research Council National Statement on Ethical Conduct in Human Research 2023 [30], the World Medical Association Declaration of Helsinki [31] and the Strengthening the Reporting of Observational Studies in Epidemiology Statement guidelines [32] (S1 Table, Additional file 1).

### Data collection

Participants provided their sociodemographic information, medical history, illness presentation and patient-reported outcomes by completing a self-administered questionnaire at three time points (T_0_, T_1_ and T_2_) separated by approximately six-month intervals. The questionnaires were distributed online, as digital offline copies, as paper copies and over the phone depending on each participant’s preference. Items capturing the participants’ sociodemographic characteristics and medical history were derived from the NCNED’s Research Registry Questionnaire, as previously described [15,24]. Symptom presentation and patient-reported outcomes (including overall health status, QoL, functional capacity and fatigue impact) were measured using existing validated and internationally recognised instruments [5,6,33–39]. Although reliability statistics are provided for the PROMs employed throughout this research, the validation of these PROMs was not a component of the present study’s analyses.

Online questionnaires were administered via the Research Electronic Data Capture (REDCap) survey platform (Vanderbilt University, Nashville, Tennessee [40,41]). All data collected via offline methods were entered into REDCap for storage. Upon commencing the first questionnaire, participants were assigned an alphanumeric code to link subsequent questionnaires and deidentify the responses in data analysis. Participants who did not submit their responses or had not been in contact with the research team within 30 days of receiving the questionnaire were considered lost to follow-up.

#### Sociodemographic characteristics.

At T_0_, participants provided their age in years, sex at birth, ethnicity, country of birth, height in centimetres, weight in kilograms and the highest level of education obtained (primary school, high school, professional training, undergraduate or postgraduate). Continuing education status and employment status were confirmed at each time point. Among those employed at the time of completing the questionnaire, the number of hours in paid employment per week and whether employment was casual, part-time or full-time were also queried. The number of hours spent completing domestic work and access of social support were requested from all participants. Social support included access to the Disability Support Pension, as well as informal care received from family or friends.

#### Medical history.

Illness onset information was collected at T_0_ to confirm the participants’ illness duration. Participants were invited to list all health concerns (including illnesses, injuries or surgeries, as well as any allergies, intolerances or sensitivities) to identify the presence of exclusionary diagnoses. “Illnesses” referred to any ongoing or resolved chronic illnesses, as well as any major acute illnesses. The T_0_ questionnaire requested all health concerns from birth to date. Subsequent questionnaires queried changes to existing health concerns and the presence of new health concerns since the previous time point. Capturing this data also permitted analysis of the presence of comorbid entities, which are described in the CCC and ICC as health conditions that are frequently concurrently diagnosed with ME/CFS [5,6]. Participants also reported the impact of their illness (yes or no) on the four life activities outlined in the ICC, including occupation, education, social activities and recreational activities, at each time point.

#### Symptom presentation.

The questionnaire captured over 50 different symptoms derived from the CCC and ICC across six symptom categories: a) neurocognitive impairments, b) pain, c) sleep disturbances, d) neurosensory, perceptual and motor disturbances, e) immune, gastrointestinal and urinary impairments, and f) energy production/transportation impairments, as well as post-exertional malaise and general malaise. Participants recorded the severity of each symptom within the month prior to completing the questionnaire, as well as whether they believed the symptom in question to be attributable to their ME/CFS, at each time point. Self-reported symptom severity was scored using the five-point Likert scale values from the Centers for Disease Control and Prevention’s Symptom Inventory for ME/CFS [37]: 1) very mild, 2) mild, 3) moderate, 4) severe or 5) very severe. Post-exertional malaise severity was scored on a three-point Likert scale: 1) a little, 2) a fair bit or 3) a lot.

Symptoms were considered present if they were reported as being: a) at least very mild within the month prior to completing the questionnaire and b) attributable to ME/CFS. Fulfilment of the CCC and ICC was ascertained by evaluating symptom presence. The Widespread Pain Index was also employed to identify participants with probable comorbid fibromyalgia (FM). Pain in at least seven body areas was considered consistent with the American College of Rheumatology’s FM case definition [39].

#### PROMs.

Validated PROMs were employed to assess the participants’ perceptions of their overall health status, QoL, functional capacity and fatigue impact. Participants’ perceptions of their overall health status were quantified with the Australia-modified Karnofsky Performance Scale (AKPS) [33] and Dr Bell’s Chronic Fatigue and Immune Dysfunction Syndrome (CFIDS) Disability Scale [34]. The scores returned by these single-item instruments correspond to the percentage of overall functioning and range from 0% to 100%, increasing in 10% increments [33,34]. QoL was assessed with the 36-Item Short-Form Health Survey version 2 (SF-36v2) [38]. Domain scores for this PROM range from 0% to 100% and correspond to the percentage of QoL [38]. Functional capacity was quantified with the World Health Organization Disability Assessment Schedule version 2.0 (WHODAS 2.0) [36]. Domain scores for this PROM range from 0% to 100% and correspond to the percentage of disability or difficulty in functioning. Fatigue impact was measured with the Modified Fatigue Impact Scale (MFIS) [35]. All domains have a minimum score of 0 with maximum scores of 36, 40 and 8 for the Physical, Cognitive and Psychosocial domains, respectively.

### Statistical analysis

This longitudinal study had a within-subjects, repeated measures design and captured categorical, Likert scale, nonparametric continuous and parametric continuous data. All data stored in REDCap was exported to Statistical Package for the Social Sciences (SPSS) version 29.0 (IBM Corp, Armonk, New York [42]) for analysis. The α-level for all statistical tests, including post-hoc analyses, was α = 0.05. All p-values are correct to two significant figures except where p < 0.001. The statistical methods employed have been guided by the existing literature [43,44] and deemed appropriate by an external biostatistician. For all variables where applicable, the number and percentage of participants with missing data are provided.

Categorical variables included the presence of additional diagnoses, the presence of comorbid entities, sex at birth, ethnicity, country of birth, education status, current employment status, access of social support, diagnostic criteria fulfilled, symptom prevalence and perceived impact on life activities. Numbers and percentages are provided for these categorical variables. Exact Cochran’s *Q* tests were used to compare the distribution of employment status, access of social support, diagnostic criteria fulfilled, symptom prevalence and perceived impact on life activities between the three time points. For categorical variables returning significance, post-hoc analyses were performed using exact McNemar’s tests for each of the three possible pairings of the study’s time points (T_0_ v T_1_, T_1_ v T_2_ and T_0_ v T_2_). Pairwise McNemar’s tests were subsequently adjusted for multiple comparisons with the Bonferroni correction.

Likert scale data included employment status among those employed, symptom severity, AKPS scores and Dr Bell’s CFIDS Disability Scale scores. Summary statistics for these data are presented as medians and consensus (C) values. Manual calculations of the consensus values were informed by Tastle *et al*. [45]. The Shapiro-Wilk test was employed to determine the normality of the continuous variables, as the size of the study sample was n < 50. Homogeneity of variances was also investigated using Levene’s test for the normally distributed variables. Normally distributed, homoscedastic continuous variables were analysed with parametric statistical analyses, whereas non-normally distributed continuous variables and normally distributed, heteroscedastic variables were compared over time using nonparametric statistical methods.

The median (M), quartile 1 to quartile 3 (Q1–Q3) and 95% confidence intervals (95%CI) of the median are provided for all nonparametric continuous variables, including body mass index (BMI), illness duration, hours of domestic work per week, all SF-36v2 domains, the Cognition, Mobility, Life Activities 2 and Participation domains of the WHODAS 2.0, and the Cognitive of the MFIS. Total number of symptoms was also treated as a nonparametric continuous variable, as the values for this variable are discrete. Friedman tests were employed to compare Likert scale data and nonparametric continuous variables across the three time points. Post-hoc analyses were conducted for variables returning significance using pairwise Wilcoxon signed-rank tests adjusted with the Bonferroni correction for multiple comparisons.

Parametric continuous variables (including age, hours of paid work per week, the Self-Care, Getting Along and Life Activities 1 domains of the WHODAS 2.0, and the Physical and Psychosocial domains of the MFIS) are summarised as means, standard deviations and 95%CIs of the mean. One-way repeated measures ANOVA models were generated to compare parametric continuous variables between the three time points. Sphericity of the parametric continuous variables was assessed with Mauchly’s test. For variables violating sphericity, the p-values adjusted with the Greenhouse-Geisser correction are reported. Outliers were also investigated to determine the validity of the ANOVA results. Post-hoc analyses were performed for variables returning significance and adjusted for multiple comparisons with the Bonferroni correction.

One-sample Wilcoxon signed-rank tests were performed to compare the SF-36v2 scores at each time point with published Australian population norms [46]. Domains returning significance were adjusted with the Benjamini-Hochberg correction for multiple comparisons.

Reliability statistics were generated for all PROM subscales where applicable. McDonald’s ω values were calculated for all PROM domains composed of at least three items at each time point to determine internal consistency, which was defined as ω ≥ 0.7 [47]. The six- and 12-month test-retest reliability of the PROM domains was investigated by performing bivariate correlations of the PROM domain scores at T_0_ when compared with those at T_1_ and T_2_. Pairwise correlations were generated with Kendall’s Tau-b for Likert-scale PROMs (including the AKPS and Dr Bell’s CFIDS Disability Scale), Spearman’s correlations for nonparametric PROM domains (including all SF-36v2 domains, the Self-Care, Getting Along and Life Activities 1 WHODAS 2.0 domains, and the Physical domain of the MFIS) and Pearson’s correlations for parametric PROM domains (including the Cognition, Mobility, Life Activities 2 and Participation WHODAS 2.0 domains and the Cognitive domain of the MFIS).

## Results

Thirty-two pwME/CFS participated at all three time points of the present longitudinal study (Fig 1). The response rate among the 246 participants who received recruitment invitations was 13.0%. Participants completed the questionnaire in a median of four (Q1–Q3 = 1–10) days at T_0_, two (Q1–Q3 = 1–10) days at T_1_ and two (Q1–Q3 = 1–8) days at T_2_. The median time to completion was 252 (Q1–Q3 = 199–302) days between T_0_ and T_1_ and 198 (Q1–Q3 = 191–210) days between T_1_ and T_2_. All sociodemographic and illness characteristics among the n = 32 participants with ME/CFS are provided in Tables 1 and 2. Comparisons between the participants with the n = 19 participants lost to follow-up and n = 18 ineligible respondents are displayed in S2 and S3 Tables (Additional files 2 and 3, respectively). An overview of the statistical analysis methods employed to compare the non-participants with the participants, as well as the results observed, is provided as supplementary material (S1 Text).

**Table 1:** Sociodemographic and illness characteristics among all study participants at T_0_.

	PwME/CFS
	(n = 32)
**Age (years, *x̄* (*s*) [95%CI])**	44.03 (10.71) [40.17–47.89]
Missing	0 (0.0)
**Sex at birth (n (%))**	
Female	22 (68.8)
Male	9 (28.1)
Missing	1 (3.1)
**Ethnicity (n (%)** ^a^	
Aboriginal Australian	2 (6.3)
Anglo-Celtic	7 (21.9)
Australian	19 (59.4)
European	5 (15.6)
Japanese	1 (3.1)
Malaysian Chinese	0 (0.0)
New Zealander	1 (3.1)
White	9 (28.1)
Missing	3 (9.4)
**Country of birth (n (%))**	
Australia	27 (84.4)
Japan	1 (3.1)
Malaysia	0 (0.0)
New Zealand	2 (6.3)
United Kingdom	1 (3.1)
Missing	1 (3.1)
**BMI (M (Q1–Q3) [95%CI])**	24.62 (21.01–29.76) [22.53–28.65]
Missing	1 (3.1)
**Education (n (%))**	
High school	7 (21.9)
Professional training (other than university)	10 (31.3)
Undergraduate	11 (34.4)
Postgraduate	4 (12.5)
Missing	0 (0.0)
**Continuing education (n (%))**	
Yes	6 (18.8)
No	25 (78.1)
Missing	1 (3.1)
**Illness duration (years, M (Q1–Q3) [95%CI])**	12.50 (6.63–20.54) [8.75–18.00]
Missing	2 (6.3)
**Comorbid entities (n (%))** ^b^	
Allergic rhinitis	10 (31.3)
Anxiety	2 (6.3)
Asthma	12 (37.5)
Chemical sensitivity	16 (50.0)
Depression	0 (0.0)
FM	29 (90.6)
Food intolerance	20 (62.5)
IBS	11 (34.4)
Irritable bladder syndrome	1 (3.1)
Migraines	5 (15.6)
Neurally mediated hypotension	5 (15.6)
Orthostatic intolerance	12 (37.5)
Postural orthostatic tachycardia syndrome	10 (31.3)
Gastro-oesophageal reflux disease^c^	6 (18.8)
Restless legs syndrome	2 (6.3)
Sensitivity to medications	12 (37.5)

Abbreviations: *95%CI* 95% confidence interval; *BMI* Body mass index; *FM* Fibromyalgia; *IBS* Irritable bowel syndrome; *M* Median; *PwME/CFS* People with Myalgic Encephalomyelitis/Chronic Fatigue Syndrome; *Q1–Q3* Quartile 1 to quartile 3. ^a^ Sum of percentages is greater than 100.0%, as some participants identified with more than one ethnicity. ^b^ Presence of frequent concurrent diagnoses with ME/CFS captured within the CCC and ICC reported at least once across the three time points. ^c^ Includes participants self-reporting a diagnosis of acid reflux or gastro-oesophageal reflux disease.

**Table 2:** Employment and social support among all study participants throughout the study.

	PwME/CFS	p
	(n = 32)	
**Currently employed (n (%))**		0.11^a^
Employed	10 (31.3)	
Discontinued employment^b^	3 (9.4)	
Unemployed	19 (59.4)	
Missing	0 (0.0)	
**Receiving Disability Support Pension (n (%))** ^c^		1.0^a^
Yes	10 (45.5)	
No	12 (54.5)	
Missing	0 (0.0)	
**Employment status (n (%))** ^d^		0.72^e^
Casual	5 (38.5)	
Part-time	11 (84.6)	
Full-time	0 (0.0)	
Missing	0 (0.0)	
**Hours of paid work per week (*x̄* (*s*) [95%CI])** ^f^		0.94^g,h^
T_0_	12.69 (9.98) [6.66–18.72]	
T_1_	12.78 (8.65) [7.56–18.01]	
T_2_	14.20 (7.63) [8.74–19.66]	
**Receiving social support (n (%))** ^i^		0.39^a^
Yes	25 (78.1)	
No	7 (21.9)	
Missing	0 (0.0)	
**Hours of domestic work per week (M (Q1–Q3) [95%CI])** ^j^		0.61^e^
T_0_	6.50 (2.00–11.00) [3.00–7.00]	
T_1_	6.00 (3.00–13.00) [4.00–10.00]	
T_2_	4.50 (2.00–14.00) [2.00–10.00]	

Abbreviations: *95%CI* 95% confidence interval; *M* Median; *NA* Not applicable; *PwME/CFS* People with Myalgic Encephalomyelitis/Chronic Fatigue Syndrome; *Q1–Q3* Quartile 1 to quartile 3. ^a^ Analysed with Cochran’s *Q* test. ^b^ Participants employed at T_0_ and T_1_ but unemployed at T_2_. ^c^ Among those who reported being unemployed at least once across the three time points (n = 22/32, 68.8%). ^d^ Among those who reported being employed at least once across the three time points (n = 13/32, 40.6%). Sum of percentages is greater than 100.0%, as some participants’ employment status changed throughout the study. ^e^ Analysed with Friedman test. ^f^ Among those who reported being employed (T_0_ n = 13/32, 40.6%; T_1_ n = 13/32, 40.6%; T_2_ n = 10/32, 31.1%). Hours of paid work refers to the maximum number of hours worked in paid employment per week reported for each time point. ^g^ Analysed with one-way repeated measures ANOVA test. As sphericity was violated, the p-value reported has been adjusted with the Greenhouse-Geisser correction. ^h^ Refers to social support being received at the time of completing the questionnaire at least once throughout the study. Social support was defined as any informal care (ranging from help with housework, cooking or shopping to full-time assistance with activities of daily living) received from family or friends. ^i^ Hours of domestic work refers to the maximum number of hours per week spent completing domestic work (such as household chores and cleaning, yard maintenance and cooking) reported for each time point. ^j^ Data missing for participants who were unsure or did not provide a response (T_0_ n = 2/32, 6.3%; T_1_ n = 3/32, 9.4%; T_2_ n = 4/32, 12.5%).

All n = 32 participants fulfilled the CCC at baseline. In addition to their ME/CFS diagnosis, ten (n = 10/32, 31.3%) participants reported additional diagnoses that they believed to be controlled. One (n = 1/32, 3.1%) participant reported diagnoses of both eosinophilic oesophagitis and localised hypermobility spectrum disorder and another (n = 1/32, 3.1%) reported diagnoses of both hydronephrosis and pulmonary foramen ovale. Other additional diagnoses included attention deficit/hyperactivity disorder (n = 1/32, 3.1%), Barrett’s oesophagus (n = 1/32, 3.1%), bipolar disorder (n = 1/32, 3.1%), hypertension (n = 1/32, 3.1%), large vein incompetence (n = 1/32, 3.1%), polycystic ovary syndrome (n = 2/32, 6.3%) and vaginal prolapse (n = 1/32, 3.1%).

### Sociodemographic and illness characteristics

Most participants were female (n = 22/32, 68.8%). The mean age and median illness duration at T_0_ was 44.03 (*s* = 10.71) years and 12.50 (Q1–Q3 = 6.63–20.54) years, respectively. All participants reported at least one comorbid entity captured within the CCC or ICC at least once across the three time points. There was a median of five (Q1–Q3 = 3–7) total comorbid entities per participant throughout the study. FM (n = 30/32, 93.8%), food intolerance (n = 20/32, 62.5%) and chemical sensitivity (n = 16/32, 50.0%) were the most common comorbid entities. Whilst only three (n = 3/32, 9.4%) participants reported having received a diagnosis of FM, 90.6% (n = 29/32) of participants returned a Widespread Pain Index score of 7 or more, consistent with the pain requirements for the American College of Rheumatology’s FM case definition [39]. Asthma (n = 12/32, 37.5%), irritable bowel syndrome (IBS) (n = 11/32, 34.4%), orthostatic intolerance (n = 12/32, 37.5%) and sensitivity to medications (n = 12/32, 37.5%) were experienced by over one-third of the participants. Additional comorbid entities reported included abnormal mast cell function (n = 2/32, 6.3%), chronic regional pain syndrome (n = 1/32, 3.1%) and vulvodynia (n = 1/32, 3.1%).

### Employment and social support

There were no significant changes in employment or social support over the 12-month study period. Most (n = 19/32, 59.4%) participants were unemployed due to their illness and remained unemployed throughout the study (p = 0.11). Over half of those unemployed (n = 12/22, 54.5%) were not receiving the Disability Support Pension at any time point (p = 1.0). One participant (n = 1/22, 4.5%) gained access to the Disability Support Pension at T_2_. Most of the employed participants (n = 11/13, 84.6%) reported working part-time at least once across the three time points (p = 0.72). None of the participants were working full-time. Three (n = 3/13, 23.1%) participants alternated between casual and part-time hours throughout the study. The mean time spent in paid work ranged from 12.69 to 14.20 hours per week (p = 0.94). Over three-quarters of the participants (n = 25/32, 78.1%) reported receiving informal care from family or friends at least once throughout the study (p = 0.39). Among these 25 participants, over half (n = 14, 56.0%) were receiving informal care at all time points. Approximately half of the participants (T_0_ n = 21/30, 65.6%; T_1_ n = 16/29, 50.0%; T_2_ n = 17/28, 53.1%) consistently spent less than 10 hours completing domestic work per week (p = 0.61).

### Illness burden

The prevalence and severity symptoms, as well as the total number of symptoms and diagnostic criteria fulfilled, throughout this study are summarised in Table 3. Distributions of symptom severity at each time point are provided in S4 Table, Additional file 4. The impacts on life activities among the participants and summary statistics for the AKPS, Dr Bell’s CFIDS Disability Scale, WHODAS 2.0 and MFIS are documented in Table 4. Summary statistics, as well as longitudinal analyses and comparisons with population norms, for the SF-36v2 domains are outlined in Table 5. Reliability statistics, including indicators of internal consistency and test-retest reliability, are provided in Table 6 for all PROMs. Most domains for which internal consistency statistics could be generated returned a sufficient ω value of greater than 0.7 at all time points. The General Health and Vitality subscales of the SF-36v2 and the Life Activities 2 subscale of the WHODAS 2.0 were the only domains to return internal consistency scores less than 0.7 at least once throughout the study (T_0_ ω = 0.684 and T_1_ ω = 0.683; T_0_ ω = 0.597; and T_0_ ω = 0.654, respectively). Scores at T_0_ were significantly correlated with those at T_1_ and T_2_ for almost all PROM domains. The Role Emotional domain of the SF-36v2 was the only domain for which neither the T_0_ and T_1_ nor the T_0_ and T_2_ pairwise correlations were significant (p = 0.18 and p = 0.77, respectively).

**Table 3:** Symptom presentation among all study participants at T_0_, T_1_ and T_2_.

	T_0_		T_1_		T_2_		p	
	(n = 32)		(n = 32)		(n = 32)			
**Total number of symptoms (M (Q1–Q3) [95%CI])**	34 (25–40) [27–38]		30 (23–43) [25–40]		33 (25–41) [27–38]		0.96^a^	
Missing	0 (0.0)		1 (3.1)		2 (6.3)			
**Diagnostic criteria (n (%))** ^b^								
CCC	32 (100.0)		27 (84.4)		29 (90.6)		0.12^c^	
ICC	22 (68.8)		20 (62.5)		21 (65.6)		1.0^c^	
Missing	0 (0.0)		1 (3.1)		0 (0.0)			
	**T_0_**		**T_1_**		**T_2_**		**p**	
	**(n = 32)**		**(n = 32)**		**(n = 32)**		**Prevalence^c^**	**Severity^a^**
	**Prevalence**	**Severity**	**Prevalence**	**Severity**	**Prevalence**	**Severity**		
	**n (%)^d^**	**M (C)^e,f^**	**n (%)^d^**	**M (C)^e,f^**	**n (%)^d^**	**M (C)^e,f^**		
**Post-exertional malaise**	32 (100.0)	3 (0.948)	30 (93.8)	3 (0.574)	31 (96.9)	3 (0.526)	1.0	0.013^g^
**Neurocognitive impairments**								
Slowed thought	31 (96.9)	3 (0.716)	31 (96.9)	3 (0.678)	28 (87.5)	3–4 (0.660)	NA	0.76
Impaired concentration	31 (96.9)	4 (0.643)	31 (96.9)	3 (0.645)	28 (87.5)	3–4 (0.642)	1.0	0.64
Confusion	27 (84.4)	3 (0.644)	25 (78.1)	2 (0.681)	25 (78.1)	3 (0.642)	1.0	0.41
Disorientation	19 (59.4)	2 (0.685)	18 (56.3)	2 (0.719)	17 (53.1)	2 (0.744)	1.0	0.56
Cognitive overload	30 (93.8)	4 (0.649)	29 (90.6)	4 (0.684)	30 (93.8)	4 (0.719)	1.0	0.96
Difficulty making decisions	29 (90.6)	3 (0.659)	28 (87.5)	3 (0.670)	27 (84.4)	3 (0.667)	0.44	0.27
Slowed speech	25 (78.1)	2 (0.661)	26 (81.3)	2 (0.647)	24 (75.0)	3 (0.709)	0.47	0.076
Impaired capacity for reading and comprehension	30 (93.8)	3 (0.623)	28 (87.5)	3 (0.747)	29 (90.6)	4 (0.631)	1.0	0.77
Short-term memory loss	28 (87.5)	3 (0.765)	28 (84.4)	3 (0.557)	26 (81.3)	3 (0.699)	1.0	0.22
**Pain**								
Headache	29 (90.6)	3 (0.678)	19 (59.4)	3 (0.756)	21 (65.6)	3 (0.641)	0.19	0.80
Migraine	9 (28.1)	3 (0.652)	10 (31.3)	3 (0.643)	9 (28.1)	3 (0.694)	1.0	0.37
Myalgia	30 (93.8)	3 (0.718)	29 (90.6)	3 (0.715)	26 (81.3)	3 (0.675)	1.0	0.030^g^
Arthralgia (without redness or swelling)	21 (65.6)	3 (0.730)	21 (65.6)	3 (0.732)	22 (68.8)	3 (0.740)	0.78	0.89
Abdominal pain	21 (65.6)	3 (0.672)	15 (46.9)	3 (0.764)	15 (46.9)	3 (0.767)	0.49	0.14
Chest pain	10 (31.3)	2 (0.739)	9 (28.1)	2 (0.717)	11 (34.4)	2 (0.721)	0.19	0.27
**Sleep disturbances**								
Insomnia	28 (87.5)	3 (0.591)	26 (81.3)	3 (0.694)	22 (68.8)	3 (0.688)	0.44	0.57
Prolonged sleep (including naps)	24 (75.0)	3 (0.802)	24 (75.0)	3 (0.665)	21 (65.6)	3 (0.794)	0.51	0.76
Sleeping most of the day and being awake at night	10 (31.3)	2 (0.551)	13 (40.6)	2 (0.601)	9 (28.1)	2 (0.617)	0.24	0.058
Frequent awakenings	25 (78.1)	3 (0.645)	18 (56.3)	3 (0.694)	20 (62.5)	3 (0.696)	0.31	0.60
Waking earlier when compared with before illness onset	11 (34.4)	3 (0.681)	8 (25.0)	3 (0.823)	8 (25.0)	3 (0.667)	0.025^g^	0.10
Vivid dreams or nightmares	12 (37.5)	3–4 (0.571)	11 (34.4)	3 (0.668)	11 (34.4)	3 (0.727)	1.0	0.44
Unrefreshed sleep	31 (96.9)	4 (0.699)	30 (93.8)	4 (0.720)	28 (87.5)	4 (0.717)	NA	0.38
**Neurosensory, perceptual and motor disturbances**								
Inability to focus vision	23 (71.9)	3 (0.601)	22 (68.8)	2–3 (0.598)	20 (62.5)	3 (0.675)	1.0	0.37
Sensitivity to sensations^h^	29 (90.6)	3 (0.643)	26 (81.3)	3 (0.640)	28 (87.5)	3 (0.756)	1.0	0.43
Impaired depth perception	15 (46.9)	3 (0.796)	13 (40.6)	3 (0.613)	12 (37.5)	3 (0.676)	1.0	0.85
Muscle weakness	28 (87.5)	3 (0.685)	26 (81.3)	3 (0.688)	27 (84.4)	3 (0.690)	1.0	0.42
Muscle twitching	16 (50.0)	2 (0.662)	17 (53.1)	2 (0.725)	14 (43.8)	2 (0.680)	0.45	0.061
Poor coordination	19 (59.4)	3 (0.681)	19 (59.4)	3 (0.623)	23 (71.9)	2 (0.665)	0.87	0.49
Feeling unsteady on feet	24 (75.0)	3 (0.573)	20 (62.5)	3 (0.641)	21 (65.6)	3 (0.687)	0.038^g^	0.44
**Immune, gastrointestinal and urinary impairments**								
Lymphadenopathy	15 (46.9)	3 (0.713)	19 (59.4)	2 (0.552)	18 (56.3)	2 (0.614)	0.40	0.40
Laryngitis	18 (56.3)	2–3 (0.686)	17 (53.1)	3 (0.619)	16 (50.0)	2 (0.758)	0.91	0.31
Sinusitis	11 (34.4)	3 (0.632)	10 (31.3)	2 (0.776)	9 (28.1)	2 (0.683)	1.0	0.82
Other flu-like symptoms	19 (59.4)	3 (0.720)	18 (56.3)	2 (0.671)	17 (53.1)	3 (0.628)	1.0	0.12
Viral infections with prolonged recovery periods	8 (25.0)	3–4 (0.627)	11 (34.4)	3 (0.816)	6 (18.8)	3 (0.893)	0.56	0.37
Nausea	16 (50.0)	3 (0.648)	15 (46.9)	3 (0.710)	16 (50.0)	3 (0.623)	1.0	0.77
Abdominal cramps	14 (43.8)	3 (0.528)	11 (34.4)	2 (0.612)	9 (28.1)	3 (0.730)	0.81	0.88
Bloating	16 (50.0)	3 (0.777)	11 (34.4)	3 (0.758)	15 (46.9)	3 (0.796)	0.44	0.55
Urinary frequency or urinary urgency	12 (37.5)	3 (0.617)	13 (40.6)	3 (0.567)	15 (46.9)	3 (0.701)	1.0	0.29
Nocturia	16 (50.0)	3 (0.771)	10 (31.3)	3 (0.661)	12 (37.5)	3 (0.812)	0.67	0.25
Sensitivities to foods, medications or chemicals	26 (81.3)	3 (0.662)	26 (81.3)	3 (0.650)	24 (75.0)	3 (0.557)	1.0	0.51
**Energy production/transportation impairments**								
Heart palpitations	16 (50.0)	3 (0.648)	12 (37.5)	3 (0.631)	15 (46.9)	3 (0.813)	1.0	1.0
Light-headedness or dizziness	26 (81.3)	3 (0.658)	19 (59.4)	3 (0.738)	20 (62.5)	3 (0.662)	0.44	0.41
Air hunger	16 (50.0)	2–3 (0.595)	15 (46.9)	3 (0.749)	13 (40.6)	3 (0.685)	0.33	0.74
Dyspnoea at rest	17 (53.1)	2 (0.705)	13 (40.6)	2 (0.668)	15 (46.9)	3 (0.580)	1.0	0.33
Exertional dyspnoea	17 (53.1)	3 (0.703)	17 (53.1)	3 (0.658)	16 (50.0)	3 (0.758)	1.0	0.88
Fatigue of chest wall muscles	9 (28.1)	3 (0.679)	13 (40.6)	2 (0.713)	14 (43.8)	2–3 (0.634)	0.24	0.37
Below normal body temperature	12 (37.5)	2 (0.692)	10 (31.3)	2 (0.705)	10 (31.3)	2 (0.718)	0.24	0.72
Sweating episodes	20 (62.5)	3 (0.739)	15 (46.9)	2 (0.743)	16 (50.0)	3 (0.823)	0.51	0.26
Recurrent feelings of feverishness	15 (46.9)	3 (0.670)	10 (31.3)	2 (0.637)	10 (31.3)	3 (0.778)	0.25	0.84
Chills	11 (34.4)	2 (0.727)	9 (28.1)	3 (0.760)	12 (37.5)	2 (0.703)	1.0	0.27
Cold extremities	15 (46.9)	3 (0.673)	17 (53.1)	3 (0.646)	16 (50.0)	3 (0.722)	0.44	0.086
Intolerance of extreme temperatures	23 (71.9)	4 (0.703)	22 (68.8)	3 (0.618)	26 (81.3)	4 (0.594)	1.0	0.10
**General malaise**	31 (96.9)	4 (0.711)	26 (81.3)	4 (0.707)	27 (84.4)	4 (0.681)	1.0	0.23

Abbreviations: *95%CI* 95% confidence interval; *C* Consensus; *CCC* Canadian Consensus Criteria; *ICC* International Consensus Criteria; *M* Median; *NA* Not applicable; *Q1–Q3* Quartile 1 to quartile 3. ^a^ Analysed with Friedman’s test. ^b^ Participants are categorised by the most stringent ME/CFS criteria fulfilled. ^c^ Analysed with Cochran’s *Q* test. ^d^ Self-reported and believed by participant to be attributable to their ME/CFS. This does not include participants with missing data, nor does this include participants who, within the month prior to completing the questionnaire and in relation to the symptom in question, did not experience it, were unsure if they had experienced it, had experienced it but were unsure if it was attributable to their ME/CFS or had experienced it but believed that it was not attributable to their ME/CFS (additional data, including the participants with missing data, is provided in S4 Table, Additional file 4). ^e^ Among those who reported experiencing the symptom in question within the month prior to completing the questionnaire and believed the symptom to be attributable to their ME/CFS. ^f^ Likert scale values: 1) very mild, 2) mild, 3) moderate, 4) severe and 5) very severe, except for post-exertional malaise: 1) a little, 2) a fair bit and 3) a lot. ^g^ Omnibus Cochran’s *Q* test was significant (p < 0.05); however, post-hoc analyses revealed no significant differences after correction for multiple comparisons. ^h^ Including sensitivity to light, noise, vibration, odour, taste or touch.

**Table 4:** Impact on life activities, overall perceptions of health status, functional capacity and fatigue impact among all study participants at T_0_, T_1_ and T_2_.

	T_0_	T_1_	T_2_	p
	(n = 32)	(n = 32)	(n = 32)	
**Impact on life activities (n (%))**				
Occupation	32 (100.0)	31 (96.9)	32 (100.0)	NA
Education^a^	23 (92.0)	23 (92.0)	25 (100.0)	1.0^b^
Education^c^	6 (100.0)	6 (100.0)	3 (75.0)	NA
Social activities	32 (100.0)	31 (96.9)	31 (96.9)	1.0^b^
Recreational activities	32 (100.0)	31 (96.9)	31 (96.9)	1.0^b^
**AKPS (M (C))** ^d^	60 (0.835)	60 (0.838)	60 (0.808)	0.94^e^
**Dr Bell’s CFIDS Disability Scale (M (C))** ^d^	30 (0.512)	30 (0.514)	30 (0.577)	0.34^e^
**WHODAS 2.0** ^f^				
Cognition (*x̄* (*s*) [95%CI])	42.50 (15.61) [36.87–48.13]	43.71 (20.37) [36.24–51.18]	42.97 (19.59) [35.91–50.03]	0.84^g^
Mobility (*x̄* (*s*) [95%CI])	60.35 (22.26) [52.33–68.38]	62.30 (20.57) [54.75–69.84]	60.55 (23.73) [51.99–69.10]	0.70^g^
Self-Care (M (Q1–Q3) [95%CI])	20.00 (7.50–40.00) [10.00–30.00]	25.00 (10.00–42.50) [20.00–40.00]	25.00 (10.00–50.00) [10.00–50.00]	0.85^e^
Getting Along (M (Q1–Q3) [95%CI])	60.00 (40.00–80.00) [50.00–70.00]	50.00 (40.00–80.00) [40.00–80.00]	55.00 (20.00–77.50) [30.00–70.00]	0.26^e^
Life Activities 1 (M (Q1–Q3) [95%CI])	75.00 (60.00–90.00) [60.00–90.00]	70.00 (50.00–100.00) [60.00–90.00]	85.00 (50.00–100.00) [50.00–100.00]	0.69^e^
Life Activities 2 (*x̄* (*s*) [95%CI])^h^	59.52 (17.05) [48.69–70.36]	58.17 (18.77) [47.33–69.00]	52.38 (31.41) [32.43–72.34]	0.52^g^
Participation (*x̄* (*s*) [95%CI])	64.20 (16.68) [57.60–70.79]	57.50 (19.65) [50.16–64.84]	56.45 (21.38) [48.61–64.29]	0.18^g^
**MFIS**				
Physical (M (Q1–Q3) [95%CI])^i^	30.00 (27.50–32.00) [28.00–32.00]	30.00 (26.00–34.00) [26.00–32.00]	30.50 (25.00–33.00) [27.00–33.00]	1.0^e^
Cognitive (*x̄* (*s*) [95%CI])^j^	26.93 (5.43) [24.87–29.00]	26.71 (7.18) [24.08–29.34]	26.45 (7.78) [23.60–29.31]	0.87^g,k^
Psychosocial (M (Q1–Q3) [95%CI])^l^	6.00 (5.00–8.00) [5.00–7.00]	6.00 (5.00–8.00) [6.00–7.00]	6.00 (5.00–7.75) [6.00–7.00]	0.19^e^

Abbreviations: *95%CI* 95% confidence interval; *AKPS* Australia-modified Karnofsky Performance Scale; *C* Consensus *CFIDS* Chronic Fatigue and Immune Dysfunction Syndrome; *M* Median; *MFIS* Modified Fatigue Impact Scale; *NA* Not applicable; *Q1–Q3* Quartile 1 to quartile 3; *WHODAS 2.0* World Health Organization Disability Assessment Schedule version 2.0. ^a^ Among those not continuing further education (T_0_ n = 25/32, 78.1%; T_1_ n = 26/32, 81.3%; T_2_ n = 27/32, 84.4%). ^b^ Analysed with Cochran’s *Q* test. ^c^ Among those continuing further education (T_0_ n = 6/32, 18.8%; T_1_ n = 6/32, 18.8%; T_2_ n = 4/32, 12.5%). ^d^ Scores correspond to the percentage of overall functioning with a minimum and maximum of 0 and 100, respectively. ^e^ Analysed with Friedman test. ^f^ Domain scores correspond to the percentage of disability or difficulty in functioning with a minimum and maximum of 0 and 100, respectively. ^g^ Analysed with one-way repeated measures ANOVA test. ^h^ Data available for participants that were employed or studying at the time of completing the questionnaire (T_0_ n = 12/32, 37.5%; T_1_ n = 14/32, 43.8%; T_2_ n = 12/32, 37.5%). ^i^ Scores correspond to the extent of fatigue impact with a minimum and maximum of 0–36, respectively. ^j^ Scores correspond to the extent of fatigue impact with a minimum and maximum of 0 and 40, respectively. ^k^ As sphericity was violated, the p-value reported has been adjusted with the Greenhouse-Geisser correction. ^l^ Scores correspond to the extent of fatigue impact with a minimum and maximum of 0 and 8, respectively.

**Table 5:** QoL among all study participants at T_0_, T_1_ and T_2_ when compared with Australian population norms.

	T_0_	T_1_	T_2_	p^a^	Population [46]	Uncorrected p^b^		
	(n = 32)	(n = 32)	(n = 32)		(n = 6,903)	T_0_ v Population	T_1_ v Population	T_2_ v Population
**SF-36v2 (M (Q1–Q3) [95%CI])** ^c^								
Physical Functioning	30.00 (20.00–38.75) [20.00–35.00]	32.50 (23.75–41.25) [25.00–35.00]	30.00 (20.00–43.75) [20.00–40.00]	0.28	95.0 (75.0–100.0) [NA]	**<0.001**	**<0.001**	**<0.001**
Role Physical	6.25 (0.00–25.00) [0.00–25.00]	12.50 (0.00–25.00) [0.00–18.75]	12.50 (0.00–18.75) [0.00–18.75]	0.97	100.0 (75.0–100.0) [NA]	**<0.001**	**<0.001**	**<0.001**
Bodily Pain	32.50 (22.50–54.38) [22.50–45.00]	45.00 (22.50–67.50) [32.50–57.50]	40.00 (22.50–57.50) [22.50–45.00]	0.35	84.0 (62.0–100.0) [NA]	**<0.001**	**<0.001**	**<0.001**
General Health	25.00 (16.67–33.33) [20.83–33.33]	25.00 (16.67–29.17) [16.67–29.17]	25.00 (13.54–32.29) [16.67–29.17]	0.22	75.0 (57.0–87.0) [NA]	**<0.001**	**<0.001**	**<0.001**
Vitality	6.25 (0.00–17.19) [0.00–12.50]	6.25 (0.00–18.75) [0.00–12.50]	6.25 (0.00–17.19) [0.00–12.50]	0.78	65.0 (50.0–80.0) [NA]	**<0.001**	**<0.001**	**<0.001**
Social Functioning	12.50 (0.00–25.00) [0.00–25.00]	12.50 (0.00–37.50) [12.50–37.50]	12.50 (0.00–25.00) [0.00–25.00]	0.49	100.0 (75.0–100.0) [NA]	**<0.001**	**<0.001**	**<0.001**
Role Emotional	79.17 (52.08–100.00) [58.33–100.00]	75.00 (66.67–100.00) [75.00–100.00]	91.67 (75.00–100.00) [75.00–100.00]	0.96	100.0 (66.7–100.0) [NA]	**<0.001**	**<0.001**	**<0.001**
Mental Health	58.33 (45.83–66.67) [50.00–66.67]	62.50 (45.83–70.83) [50.00–70.83]	62.50 (51.04–70.83) [54.17–70.83]	0.089	80.0 (68.0–88.0) [NA]	**<0.001**	**<0.001**	**<0.001**

Abbreviations: *95%CI* 95% confidence interval; *M* Median; *NA* Not applicable; *Q1–Q3* Quartile 1 to quartile 3; *QoL* Quality of life; *SF-36v2* 36-Item Short-Form Health Survey version 2. Bolded p-values indicate significance (p < 0.05) after correction for multiple comparisons. ^a^ Analysed with Friedman’s test. ^b^ Analysed with one-sample Wilcoxon signed-rank test. ^c^ Domain scores correspond to the percentage of QoL with a minimum and maximum of 0 and 100, respectively.

**Table 6:** Complete reliability statistics of the PROM domains among all study participants throughout the study.

	Internal consistency (ω)			Test-rest reliability			
	T_0_	T_1_	T_2_	T_0_ v T_1_		T_0_ v T_2_	
	(n = 32)	(n = 32)	(n = 32)	*r*	p	*r*	p
**AKPS**	NA^a^	NA^a^	NA^a^	0.623^b^	**<0.001**	0.416^b^	**0.0051**
**Dr Bell’s CFIDS Disability Scale**	NA^a^	NA^a^	NA^a^	0.597^b^	**<0.001**	0.550^b^	**<0.001**
**SF-36v2**							
Physical Functioning	NA^c^	0.878	0.915	0.860^d^	**<0.001**	0.685^d^	**<0.001**
Role Physical	0.812	0.838	0.925	0.380^d^	**0.038**	0.555^d^	**<0.001**
Bodily Pain	NA^a^	NA^a^	NA^a^	0.608^d^	**<0.001**	0.498^d^	**0.0037**
General Health	0.684	0.683	0.813	0.516^d^	**0.0042**	0.698^d^	**<0.001**
Vitality	0.597	0.748	0.861	0.645^d^	**<0.001**	0.750^d^	**<0.001**
Social Functioning	NA^a^	NA^a^	NA^a^	0.529^d^	**0.0027**	0.465^d^	**0.0084**
Role Emotional	0.956	0.889	0.947	0.251^d^	0.18	0.053^d^	0.77
Mental Health	0.851	0.871	0.830	0.738^d^	**<0.001**	0.603^d^	**<0.001**
**WHODAS 2.0**							
Cognition	0.808	0.870	0.894	0.765^e^	**<0.001**	0.521^e^	**0.0022**
Mobility	0.853	0.810	0.858	0.734^e^	**<0.001**	0.731^e^	**<0.001**
Self-Care	0.874	0.862	0.864	0.842^d^	**<0.001**	0.696^d^	**<0.001**
Getting Along	0.808	0.834	0.878	0.784^d^	**<0.001**	0.573^d^	**<0.001**
Life Activities 1	0.902	0.895	0.940	0.676^d^	**<0.001**	0.542^d^	**0.0014**
Life Activities 2	0.654	0.740	0.977	0.650^e^	**0.022**	0.332^e^	0.35
Participation	0.808	0.872	0.858	0.768^e^	**<0.001**	0.601^e^	**<0.001**
**MFIS**							
Physical	NA^c^	0.796	0.870	0.837^d^	**<0.001**	0.778^d^	**<0.001**
Cognitive	0.819	0.930	0.935	0.744^e^	**<0.001**	0.261^e^	0.18
Psychosocial	NA^a^	NA^a^	NA^a^	0.735^d^	**<0.001**	0.414^d^	**0.020**

Abbreviations: *AKPS* Australia-modified Karnofsky Performance Scale; *CFIDS* Chronic Fatigue and Immune Dysfunction Syndrome; *MFIS* Modified Fatigue Impact Scale; *NA* Not applicable; *PROM* Patient-reported outcome measure; *SF-36v2* 36-Item Short-Form Health Survey version 2; *WHODAS 2.0* World Health Organization Disability Assessment Schedule version 2.0. Bolded values indicate significance (p < 0.05). ^a^ Internal consistency statistics could not be generated, as a minimum of three items is required to calculate an ω value. ^b^ Kendall’s Tau-b correlation coefficient. ^c^ An ω value could not be calculated, as covariance was negative or equal to 0. ^d^ Spearman’s correlation coefficient. ^e^ Pearson’s correlation coefficient.

#### Symptom presentation.

The median number of symptoms within the month prior to completing the questionnaire ranged from 30 to 34 at each time point. At all three time points, at least one-quarter of the participants reported experiencing over 40 symptoms. The highest number of symptoms experienced at any time point was 53 (T_1_ n = 1/32, 3.1%). No significant changes were observed in the total number of symptoms over the 12-month study period (p = 0.96). Symptom data were missing for n = 1 participant at T_1_ who otherwise fulfilled the CCC and ICC at the first and final time points.

Importantly, none of the 54 symptoms queried in this longitudinal study were significantly different in prevalence or severity between the three time points. P-values could not be generated for the comparisons of slowed thought or unrefreshed sleep prevalence, as all participants providing valid data had consistently experienced these symptoms within the month prior to completing the questionnaire (T_0_ n = 31/31, 96.9%; T_1_ n = 31/31, 96.9%; T_2_ n = 28/28, 87.5% and T_0_ n = 31/31, 96.9%; T_1_ n = 30/30, 93.8%; T_2_ = 28/28, 87.5%, respectively). Four symptom variables returned significant omnibus tests: the severity of post-exertional malaise, severity of myalgia, prevalence of waking earlier when compared with before illness onset and prevalence of feeling unsteady on feet. However, significance was lost for these symptoms upon adjustment for multiple comparisons.

Post-exertional malaise was experienced by all participants providing valid data at T_0_ and T_1_ (n = 32/32, 100.0%; n = 30/30, 93.8%, respectively). One participant (n = 1/32, 3.1%) reported not experiencing post-exertional malaise at T_2_. Median post-exertional malaise severity scores corresponded to the highest level of post-exertional malaise severity at all time points. Consensus values decreased for post-exertional malaise severity scores from T_0_ (C = 0.948) to follow-up (T_1_ C = 0.574; T_2_ C = 0.526). Although the distribution of post-exertional malaise severity appeared to broaden throughout the study, at least 70% of all participants providing valid data reported the highest level of post-exertional malaise severity at each time point.

Thirty-four symptoms consistently returned a median severity score of at least moderate throughout the study. In the pain and sleep disturbances categories, all but one symptom returned a median of moderate severity at each time point. Eleven symptoms had a prevalence of more than 80% at all time points, six of which were neurocognitive impairments. These included slowed thought, impaired concentration, cognitive overload, difficulty making decisions, impaired capacity for reading and comprehension, and short-term memory loss, as well as myalgia, unrefreshed sleep, sensitivity to sensations, muscle weakness and general malaise. Impaired concentration, cognitive overload, impaired capacity for reading and comprehension, unrefreshed sleep and general malaise were reported as being severe by 50% or more of the study cohort at least once throughout the study. The only other symptom returning a median severity score of severe was intolerance of extreme temperatures.

Hence, the most burdensome symptoms — being those consistently experienced by greater than 80% of the study cohort and returning a median severity score of severe — were post-exertional malaise, cognitive overload, impaired capacity for reading and comprehension, unrefreshed sleep and general malaise. Among the participants providing valid data, over one-third and one-quarter experienced very severe unrefreshed sleep and general malaise, respectively, at each time point.

#### Impact on life activities.

Almost all participants reported impacts on life activities throughout the study with no significant changes over the 12-month study period. Participation in one’s occupation, social activities and recreational activities were restricted due to illness among all participants providing valid data at T_0_ (n = 32/32, 100.0%; n = 32/32, 100.0%; n = 32/32, 100.0%, respectively) and T_1_ (n = 31/31, 96.9%; n = 31/31, 96.9%; n = 31/31, 96.9%, respectively). Similar findings were observed at T_2_ except that one participant (n = 1/32, 3.1%) reported no impact on social or recreational activities. The ability to pursue education was consistently limited for all participants continuing further education and over 90% of the total study cohort.

#### Overall perceptions of health status.

Considerably poor scores were returned for both the AKPS and Dr Bell’s CFIDS Disability Scale with medians of 60% and 30%, respectively, consistent throughout the study. Consensus values for the Dr Bell’s CFIDS Disability Scale scores (T_0_ C = 0.512; T_1_ C = 0.514; T_2_ C = 0.577) appeared lower than those for the AKPS (T_0_ C = 0.835; T_1_ C = 0.838; T_2_ C = 0.808). This may be attributed to the AKPS capturing a broader range of health states. There were no significant changes over the 12-month study period for either scale.

#### QoL.

**Fig 2:**
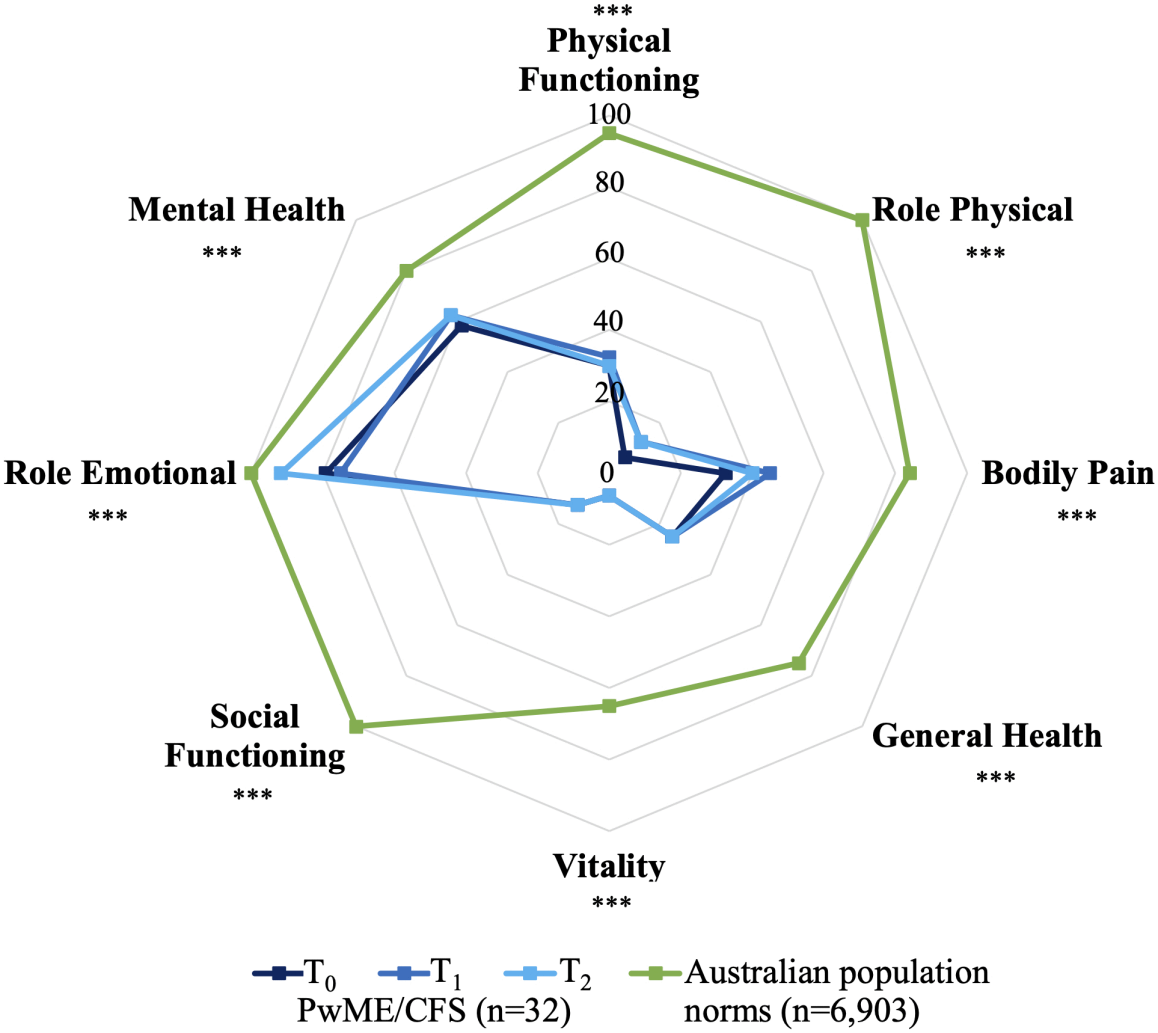
Median SF-36v2 scores among all study participants over the 12-month study period when compared with Australian population norms. Abbreviations: *PwME/CFS* People with Myalgic Encephalomyelitis/Chronic Fatigue Syndrome; *SF36v2* 36-Item Short-Form Health Survey version 2. Australian population norms extracted from Stevenson *et al*. [46]. The centre score represents the minimum possible score for this scale (0%) and corresponds to the poorest QoL, whereas the score on the outer gridline is the maximum possible score for this scale (100%) and indicates the highest QoL. Omnibus p-values for the comparisons of SF-36v2 scores among the study participants across the three time points are provided in Table 5. *** Adjusted p < 0.001 at all time points when compared with population norms.

None of the SF-36v2 domain scores differed significantly throughout the study (Fig 2). The study participants returned significantly impaired scores for all SF-36v2 domains when compared with population norms at all three time points (all p < 0.001, adjusted). Vitality, Role Physical and Social Functioning were the most impacted SF-36v2 domains with medians consistently ranging from 6.25% to 12.50%. Additionally, over 90% of the study cohort returned Vitality, Role Physical and General Health scores of less than 50% at each time point. Although significantly impaired when compared with population norms, median Role Emotional and Mental Health scores were the highest of the SF-36v2 domains and ranged from 75.00% to 91.67% and 58.33% to 62.50%, respectively.

#### Functional capacity.

**Fig 3:**
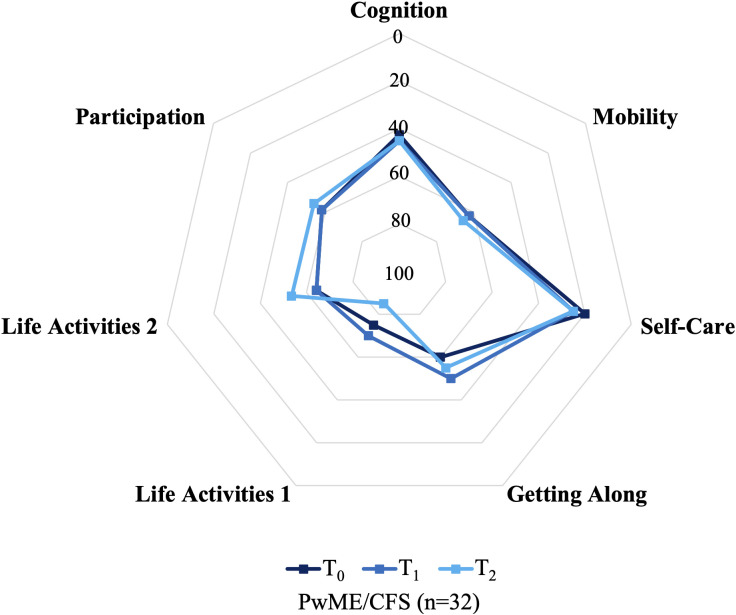
Median WHODAS 2.0 scores among all study participants over the 12-month study period. Abbreviations: *PwME/CFS* People with Myalgic Encephalomyelitis/Chronic Fatigue Syndrome; *WHODAS 2.0* World Health Organization Disability Assessment Schedule version 2.0. The centre score is the maximum possible score for this scale (100%) and corresponds to the highest disability, whereas the score on the outer gridline is the minimum possible score for this scale (0%), which indicates the lowest disability. Omnibus p-values for the comparisons of WHODAS 2.0 scores across the three time points are provided in Table 4.

Life Activities 1, Mobility and Participation were the most impacted WHODAS 2.0 domains (Fig 3). This aligns with the significantly lower scores observed in the Vitality and Role Physical domains of the SF-36v2. Median scores were higher for Life Activities 1 when compared with the other WHODAS 2.0 domains and ranged from 70% to 85% throughout the study. Additionally, around one-quarter of participants reported 100% disability for this domain at each time point. All WHODAS 2.0 domains returned a mean or median score greater than 50% except Cognition and Self-Care. Nevertheless, a score of at least 50% was reported by approximately one-third and one-quarter of the participants for Cognition and Self-Care, respectively, at each time point and the highest scores for these domains ranged from 90% to 100% throughout the study. Importantly, there were no significant differences between the time points for any of the WHODAS 2.0 domains.

#### Fatigue impact.

**Fig 4:**
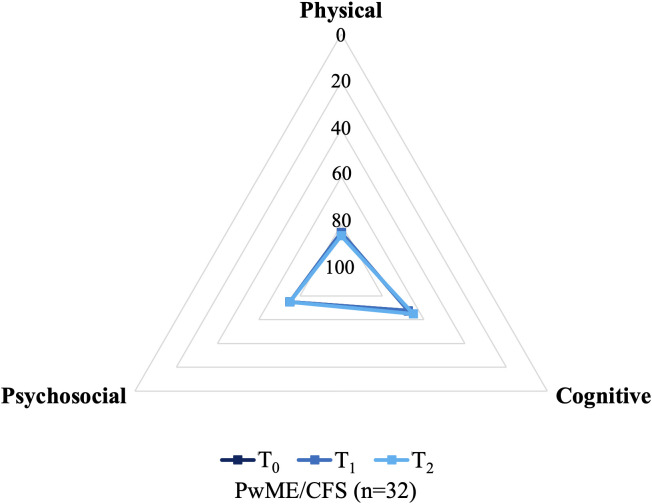
Median MFIS scores among all study participants over the 12-month study period. Abbreviations: *PwME/CFS* People with Myalgic Encephalomyelitis/Chronic Fatigue Syndrome; *MFIS* Modified Fatigue Impact Scale. The median MFIS scores for each time point have been converted to percentages of the maximum possible score for the corresponding domain to allow the three subscales to be presented on the same scale. Minimum and maximum values for the Physical, Cognitive and Psychosocial domains are 0 to 36, 0 to 40 and 0 to 8, respectively. The maximum score is presented at the centre, whereas the minimum score is situated on the outer gridline. Omnibus p-values for the comparisons of WHODAS 2.0 and MFIS scores across the three time points are provided in Table 4.

Similar to the results returned by the SF-36v2 and WHODAS 2.0, the Physical domain of the MFIS was the most impacted (Fig 4). Median Physical scores ranged from 30.00 to 30.50 throughout the study, representing approximately 80% of total fatigue impact in this domain. Psychosocial followed Physical as the second-most impacted MFIS domain, with a median score of 6 at each time point corresponding to 75% of total fatigue impact. Over 90% of the participants reported a score greater than 50% for both the Physical and Psychosocial MFIS domains at each time point. Mean Cognitive scores ranged from 26.45 to 26.93 throughout the study, indicating approximately 65% of total fatigue impact. Around three-quarters of the participants reported a score greater than 50% for the Cognitive domain. Fatigue impact was not significantly different in any of the three domains over the 12-month study period.

## Discussion

The present longitudinal investigation provides evidence that the impacts of living with ME/CFS on the health and wellbeing of those affected are profound and prolonged, thereby affirming the largely permanent nature of the condition. There is a paucity of research reporting the impacts of living with ME/CFS on QoL among those affected over time [48]. Furthermore, in the Australian context, no longitudinal patient-reported outcome data has been published among pwME/CFS meeting the CCC or ICC prior to the present study. The detailed illness presentation and patient-reported outcome data reported herein, therefore, provide a novel, comprehensive evaluation of the impacts of ME/CFS on those affected in Australia. By expanding upon existing Australian research [12,15,16,24,25], this longitudinal investigation may guide the reform of national healthcare policy to reflect the lived experiences and support needs of pwME/CFS.

Healthcare policies that are guided by consumers’ lived experiences facilitate the implementation of person-centred healthcare services [49–52]. Such person-centred care that has been informed through consumer engagement and direct involvement of consumers in their care optimises health outcomes by prioritising consumers’ health goals and supporting positive interactions with healthcare providers and the healthcare system [49–52]. By providing novel, detailed data characterising the profound and protracted illness burdens faced by Australians with ME/CFS, the findings of the present study and the lived experiences documented herein serve to inform the development of evidence-based, person-centred healthcare policies for ME/CFS in Australia. This is vital to ensure that the care needs of pwME/CFS are met, which is not only paramount to improve health outcomes for those affected but also to avoid further deterioration in health caused by limited access to necessary care.

Participants repeatedly experienced substantial illness burdens throughout this study. Comorbid entities requiring targeted treatment (such as FM, IBS, asthma and orthostatic issues) were common. Post-exertional malaise, neurocognitive impairments, unrefreshed sleep and general malaise were consistently the most burdensome symptoms. This corroborates the findings reported in an Australian longitudinal study among pwME/CFS meeting the CCC published in 2021 by Balinas *et al*. [12]. The present study expanded upon the observations of Balinas *et al*. [12] by providing symptom presentation variables in novel detail, including symptom presence, symptom severity and the most extensive list of individual symptoms to date in Australian ME/CFS research. It should also be noted that data were collected by Balinas *et al*. [12] in the first year of the pandemic. During this time, poorer health outcomes were reported across the Australian population [53,54]. Nevertheless, the findings of Balinas *et al*. [12] are replicated in the present study. Similarly, the QoL scores reported herein are comparable with those of a pre-pandemic Australian ME/CFS cohort fulfilling the ICC published by Johnston *et al*. [25] in 2014. This emphasises that, irrespective of external pressures, the debilitating burden and disability faced by pwME/CFS remains ongoing and valid.

Most PROM domains consistently returned a median score below 50% of QoL or functioning, indicating a substantial and widespread impact on participants’ health and wellbeing. All SF-36v2 domains were significantly impaired when compared with population data, with most domains scoring lower than the 25^th^ percentiles documented among the Australian population [46]. Noteworthy impairments were observed in the Role Physical and Vitality domains of the SF-36v2, the Life Activities 1 of the WHODAS 2.0 and the Physical domain of the MFIS. These findings indicate extensive disability and difficulty in completing daily and work life activities. Profound impairments in these domains have been repeatedly observed in other studies [15,55] and the trends documented in the present study corroborate the findings of a systematic review of nine publications examining HRQoL among pwME/CFS fulfilling the CCC or ICC when compared with healthy controls [48]. The median AKPS scores among pwME/CFS in the present study are also consistent with the existing literature [48] and are reminiscent of the functional status of people in palliative care [33,56].

Importantly, no significant differences were observed in any measure of symptom presentation, overall perceptions of health status, QoL, functional capacity or fatigue impact over the 12-month study period. Hence, the present study highlights that the profound impacts on the health and wellbeing of pwME/CFS are persistent and long-term. Moreover, this study captured the lived experiences of pwME/CFS with established illness, as most participants had lived with a diagnosis of ME/CFS for at least one decade.

Whilst there were no significant changes over the 12-month study period, fluctuations in employment status, access to informal care, the diagnostic criteria fulfilled, symptoms and patient-reported outcomes were observed. Assistance from family members or friends was required by 78% of the study cohort at least once but changed throughout the study for approximately one-third of participants. It is common for the presentation of ME/CFS to be fluid, with fluctuations in symptoms over time [2,8,18,57]. Such fluctuations in functioning, however, further complicate the fulfilment of permanency requirements (in addition to the lack of a biological indicator of ME/CFS), as current disability assessments consider one’s typical illness presentation and impairments at a single time point (being when the assessment is completed) [20].

Nevertheless, recovery from ME/CFS is rare — occurring in less than 10% of cases [1,8–10]. It must also be acknowledged that, whilst the functional limitations and care needs of pwME/CFS can be fluid [1,7,11,18,20,57], these fluctuations are within the boundaries of the condition, which exerts a constant and substantial burden, as evidenced in the present study. Whilst improvement may be initially observed after onset, pwME/CFS typically experience a plateauing of their symptoms as the illness progresses, which may remain stable or further deteriorate over time [1,2,8,18,57]. The prognosis of ME/CFS remains incompletely studied and is complicated by the absence of a clinical biomarker proportionate to illness severity [6,57]. However, epidemiological studies report that between 15% and 20% of pwME/CFS gradually deteriorate and approximately 60% to 70% of pwME/CFS experience either persistent or fluctuating symptoms that neither substantially improve nor worsen over time [1,2,8,18,57].

Despite this, Australian disability assessments do not accommodate the nuanced illness presentation experienced by pwME/CFS and continue to gate keep necessary support services by enforcing inappropriate eligibility requirements on this illness cohort [11,20]. Specifically, the requirement for biological evidence to confirm disability permanency must be removed from disability assessments among pwME/CFS. None of the existing published and validated case definitions that are recommended by international guidelines for the diagnosis of ME/CFS support the use of any biological indicator to confirm a diagnosis of the condition [1,2,5–8]. The present study employed a suite of validated PROMs (which are used routinely in epidemiological research internationally to evaluate health status and functional capacity) to comprehensively assess disability among pwME/CFS, identifying profound, widespread impairments not only consistent with but exceeding the minimum duration requirement of six months in the Australian Public Service Commission’s [26] definition of disability. This is further supported by the participants’ median illness duration of over one decade. In the case of ME/CFS, the requirement for biological evidence of illness presence and permanency may be substituted with the fulfilment of the most stringent diagnostic criteria for ME/CFS that is currently available, being the CCC and ICC. These case definitions are the preferred means of ME/CFS diagnosis in the continuing absence of an illness-specific biomarker [1,2,5–8]. The present study suggests that the use of these case definitions has a low likelihood of false positives, as a small number of the participants (who were experiencing formally diagnosed illness persisting for at least one decade) fluctuated out of the diagnostic criteria briefly despite their extensive symptom burden. For this reason, policies governing access to disability and social support services in Australia must also be reformed to enable the extent of disability among pwME/CFS to be assessed over multiple time points.

Additionally, longitudinal evaluations must be incorporated into disability assessments among pwME/CFS to capture the impacts of post-exertional malaise. Current disability assessments do not consider the implications of post-exertional malaise, which is an essential component of the illness presentation of ME/CFS [20]. For pwME/CFS, the ability to complete activities encompasses current functioning, as well as the repercussions of exertion [7,8,11,20,58]. Functional limitations due to post-exertional malaise must be captured within disability assessments using validated measures and across sufficient time periods to fairly examine disability in pwME/CFS [20].

Whilst the present study reiterates the long-term, disabling nature of ME/CFS and emphasises the need for improved access to disability and social support services, data identifying the specific care and support needs of pwME/CFS in Australia is sparse [12,14]. Further research must document the care needs of pwME/CFS to guide tailored service planning. Additionally, future longitudinal investigations should be expanded to capture other chronic multi-systemic illness, such as Long COVID and Gulf War Illness [59,60], to inform healthcare policies and care protocols with illness-specific data.

The present longitudinal research project forms part of a larger research plan that continues to monitor clinical outcomes and QoL among pwME/CFS. This ongoing research serves to expand the Australian ME/CFS literature to aid in monitoring the burden of ME/CFS on Australians with the condition, as well as the Australian healthcare system. Data reported in this current investigation have been disseminated through our community communication models. These namely include local, state and national ME/CFS Patient Associations (totalling approximately 10,000 community stakeholders). The findings of this research will also be distributed via our international community communication models, such as social media, where our metrics report in excess of 20,000 stakeholders (nationally and internationally) and the International ME Chronicle that has a readership base in excess of 30,000 stakeholders.

### Strengths and limitations

This longitudinal investigation benefitted from a suite of validated and internationally recognised PROMs to capture the multifactorial impacts of ME/CFS. The use of multiple modalities to distribute the questionnaires enabled flexibility in participation, serving to reduce volunteer bias and allowing people with severe illness to participate in the study. Additionally, collecting symptom presentation and patient-reported outcome data within the month prior to completing the questionnaire rather than at the time of questionnaire completion mitigated bias due to fluctuations in functional status. As all the sociodemographic and illness characteristics, as well as most of the employment and social support characteristics, were comparable between the participants and non-participants when controlling for confounders and adjusting for multiple comparisons, the results observed among the present study cohort of pwME/CFS were not substantially skewed by volunteer biases. However, the small sample size of the present longitudinal study — which is an inherent limitation of epidemiological research among pwME/CFS [48] — potentially compromises the generalisability of these findings, particularly for pwME/CFS belonging to marginalised populations. Large-scale longitudinal studies are paramount to further support the findings of the present investigation.

All pwME/CFS met the most stringent diagnostic criteria available (being the CCC and ICC), which are the recommended means of diagnosis in the absence of a laboratory-based test by international guidelines and peak public health bodies [1,2,5–8], thereby ensuring that the results observed were attributable to ME/CFS. Additionally, participants with comorbidities that may compound the impacts on health and wellbeing were excluded from the study. Nevertheless, as additional diagnoses are common among pwME/CFS [1,8,61], participants reporting controlled, non-exclusionary conditions that were not largely responsible for their illness presentation were included in this study to capture the breadth of lived experiences among pwME/CFS. Symptoms were required to be both experienced within the month prior to the questionnaire and attributed by the participant to their ME/CFS to ensure the prevalence of symptoms was not inflated by additional diagnoses, medication side effects, or acute illnesses or injuries that occurred within the study period.

The findings documented herein provide insight into the long-term illness burdens among Australians with ME/CFS in novel detail. This publication, therefore, begins to address the lacunae in the existing Australian ME/CFS literature. However, this study followed a small convenience sample and the response rate among the sampling frame was relatively low (13.0%). Low response rates have similarly been observed in other epidemiological studies [62,63] and are likely attributable to the restrictions that ME/CFS imposes on the ability of people with the condition to participate in research. Nevertheless, 62.7% of the participants who completed the T_0_ questionnaire were retained for the entirety of the study. Most of these participants were female and middle-aged, consistent with the existing literature [4,15,48]. Combined with the absence of significant differences in symptom presentation and patient-reported outcomes throughout the study, this indicates that the findings of the present study are largely generalisable to the population of pwME/CFS fulfilling the CCC and ICC. The data documented within this manuscript, therefore, address the significant gaps in the literature that pose a critical barrier to the development of evidence-based policies for pwME/CFS in Australia. Expanded primary care data sharing and the development of a national ME/CFS registry should be pursued to facilitate improved surveillance of health outcomes among the Australian ME/CFS population.

All statistical methods chosen were selected based on the distribution and nature of the data, as guided by the existing literature [43,44] and confirmed by an external biostatistician. This aided in reducing biases in the analyses and reporting of results. However, whilst the statistical methods chosen were suitable for the data’s characteristics, these tests limited analysis of potential confounding variables (such as age, sex at birth and illness duration) in longitudinal comparisons of symptom presentation. Future research among larger sample sizes should employ mixed-effects regression models to enable the inclusion of covariates.

Furthermore, the presence of comorbid entities in the present study was determined from the participants’ anamneses and was not specifically queried. Consequently, the prevalence of these conditions may be underreported in this study cohort. Additionally, whilst this study compared the participants’ QoL scores with the most recent publicly available population norms, these data were published in 1996 [46]. These scores likely do not capture the current QoL of the Australian population, including the impacts attributable to the pandemic [53,54]. The present study also described the ethnicity and country of birth of the study participants, which are often omitted in publications documenting impacts among pwME/CFS [48]. However, there were few participants belonging to culturally, linguistically or gender diverse populations. Hence, the present study cohort may not be representative of the broader ME/CFS population in Australia. Future research is required to understand the specific illness burdens and care needs for subpopulations of Australians with ME/CFS.

## Conclusions

This novel longitudinal study exemplifies the profound and prolonged illness burdens experienced by pwME/CFS and provides the necessary evidence to leverage change to Australian healthcare policies. Importantly, no significant differences were observed in any symptom or patient-reported outcome over the 12-month study period. The consistent, substantial impairments in health and wellbeing (particularly physical health and the ability to complete daily and work life activities) among pwME/CFS, as observed in this study, provide evidence for illness permanency. Reforms to Australian healthcare policies that govern access to disability and social support services must be prioritised to ensure necessary care is delivered to people with this serious, life-long illness. Revising disability assessments to appropriately capture the debilitating impairments experienced by pwME/CFS will be vital in improving service accessibility. Addressing these care inequities for pwME/CFS is essential to maximise their QoL and ability to participate in life and their communities.

## Supporting information

S1 Table Strengthening the reporting of observational studies in epidemiology statement checklist for cohort studies [32]. Abbreviations: NA Not applicable. ^a^ Give information separately for exposed and unexposed groups.
(XLSX)

S2 Table Sociodemographic and illness characteristics of the non-participants when compared with the study participants. Abbreviations: *95%CI* 95% confidence interval; *BMI* Body mass index; *FM* Fibromyalgia; *IBS* Irritable bowel syndrome; *M* Median; *PwME/CFS* People with Myalgic Encephalomyelitis/Chronic Fatigue Syndrome; *Q1–Q3* Quartile 1 to quartile 3. Bolded values indicate that at least one post-hoc test returned significance (p < 0.05) after correction for multiple comparisons. Data was collected from the ineligible respondents during screening. ^a^ Analysed with independent samples one-way ANOVA test. ^b^ Analysed with the Fisher-Freeman-Halton test. ^c^ Sum of percentages is greater than 100.0% for each cohort, as some participants identified more than one ethnicity. ^d^ Analysed with the Chi-square test. ^e^ Analysed with the Kruskal-Wallis *H* test. ^f^ Presence of frequent concurrent diagnoses with ME/CFS captured within the CCC and ICC reported at least once across the three time points for the n = 32 pwME/CFS, at T_0_ for the n = 19 participants lost to follow-up or at screening for the n = 18 ineligible respondents. ^g^ Includes participants self-reporting a diagnosis of acid reflux or gastro-oesophageal reflux disease. * Ineligible respondents < pwME/CFS ** Ineligible respondents < participants lost to follow-up *** Participants lost to follow-up <pwME/CFS ^†^ PwME/CFS < ineligible respondents.
(XLSX)

S3 Table Employment and social support among the non-participants when compared with the study participants. Abbreviations: *95%CI* 95% confidence interval; *M* Median; *NA* Not applicable; *PwME/CFS* People with Myalgic Encephalomyelitis/Chronic Fatigue Syndrome; *Q1–Q3* Quartile 1 to quartile 3. Bolded values indicate that at least one post-hoc test returned significance (p < 0.05) after correction for multiple comparisons. Data was collected from the ineligible respondents during screening. ^a^ Analysed with the Chi-square test. ^b^ Participants employed at T_0_ and T_1_ but unemployed at T_2_. ^c^ Among those unemployed at T_0_ (among the participants lost to follow-up) or at screening (among the ineligible respondents). ^d^ Analysed with the Fisher-Freeman-Halton test. ^e^ Among those employed at T_0_ (among the participants lost to follow-up) or at screening (among the ineligible respondents). ^f^ Omnibus Fisher-Freeman-Halton test was significant (p < 0.05); however, post-hoc analyses revealed no significant differences after correction for multiple comparisons. ^g^ Analysed with independent samples one-way ANOVA test. Data missing for employed participants who were unsure or did not provide a response (participants lost to follow-up n = 1/8, 12.5%; ineligible respondents n = 1/10, 10.0%). ^h^ Refers to social support being received at the time of completing the questionnaire. Social support was defined as any informal care (ranging from help with housework, cooking or shopping to full-time assistance with activities of daily living [such as eating or showering]) received from family or friends. ^i^ Hours of domestic work refers to the maximum number of hours per week spent completing domestic work (such as household chores and cleaning, yard maintenance and cooking) reported at T_0_ (among the participants lost to follow-up) or at screening (among the ineligible respondents). ^j^ Analysed with the Kruskal-Wallis *H* test. Data missing for participants who were unsure or did not provide a response (participants lost to follow-up n = 6/19, 31.6%; ineligible respondents n = 4/18, 22.2%). * PwME/CFS < participants lost to follow-up.
(XLSX)

S4 Table Complete distribution statistics of symptom severity among all study participants at T_0_, T_1_ and T_2_. The prevalence and severity of all symptoms are self-reported and, for responses other than “none” or “no response”, are believed by the participants to be attributable to their ME/CFS. ^a^ In relation to the symptom in question, this category includes participants who, within the month prior to completing the questionnaire, did not experience it, were unsure if they had experienced it, had experienced it but were unsure if it was attributable to their ME/CFS or had experienced it but believed that it was not attributable to their ME/CFS. ^b^ Including sensitivity to light, noise, vibration, odour, taste or touch.
(XLSX)

S1 Text Comparisons between participants and non-participants [64,65]. (DOCX)

## Abbreviations

95%CI: 5% confidence interval
AKPS: Australia-modified Karnofsky Performance Scale
BMI: Body mass index
C: Consensus
CCC: Canadian Consensus Criteria
CFIDS: Chronic Fatigue and Immune Dysfunction Syndrome
COVID(-19): Coronavirus Disease (2019)
FM: Fibromyalgia
IBS: Irritable bowel syndrome
ICC: International Consensus Criteria
M: Median
ME/CFS: Myalgic Encephalomyelitis/Chronic Fatigue Syndrome
MFIS: Modified Fatigue Impact Scale
NA: Not applicable
NCNED: National Centre for Neuroimmunology and Emerging Diseases
PROM: Patient-reported outcome measure
PwME/CFS: People with Myalgic Encephalomyelitis/Chronic Fatigue Syndrome
Q1–Q3: Quartile 1 to quartile 3
QoL: Quality of life
REDCap: Research Electronic Data Capture
SF-36v2: 36-Item Short-Form Health Survey version 2
SPSS: Statistical Package for the Social Sciences
WHODAS 2.0: World Health Organization Disability Assessment Schedule version 2.0

## References

[1] Baraniuk JN, Marshall-Gradisnik S, Eaton-Fitch N. Myalgic Encephalomyelitis (Chronic Fatigue Syndrome). BMJ Best Practice. 2024. https://bestpractice.bmj.com/topics/en-us/277

[2] Friedberg F, Bateman L, Bested AC, Davenport T, Friedman KJ, Gurwitt A. Chronic Fatigue Syndrome/Myalgic Encephalomyelitis — Primer for Clinical Practitioners. 2014 ed. 2014.

[3] Carruthers BM, Van de Sande MI, De Meirleir KL, Klimas NG, Broderick G, Mitchell T. Myalgic Encephalomyelitis — Adult and paediatric: International consensus primer for medical practitioners. 2012.

[4] Lim E-J, Ahn Y-C, Jang E-S, Lee S-W, Lee S-H, Son C-G. Systematic review and meta-analysis of the prevalence of chronic fatigue syndrome/myalgic encephalomyelitis (CFS/ME). J Transl Med. 2020;18(1):100. doi: 10.1186/s12967-020-02269-0 32093722 PMC7038594

[5] Carruthers BM, Jain AK, De Meirleir KL, Peterson DL, Klimas NG, Lerner AM, et al. Myalgic Encephalomyelitis/Chronic Fatigue Syndrome. J Chronic Fatigue Syndr. 2003;11(1):7–115. doi: 10.1300/j092v11n01_02

[6] Carruthers BM, Van de Sande MI, De Meirleir KL, Klimas NG, Broderick G, Mitchell T, et al. Myalgic encephalomyelitis: International Consensus Criteria. J Intern Med. 2011;270(4):327–38. doi: 10.1111/j.1365-2796.2011.02428.x 21777306 PMC3427890

[7] Bested AC, Marshall LM. Review of Myalgic Encephalomyelitis/Chronic Fatigue Syndrome: an evidence-based approach to diagnosis and management by clinicians. Rev Environ Health. 2015;30(4):223–49. doi: 10.1515/reveh-2015-0026 26613325

[8] Bateman L, Bested AC, Bonilla HF, Chheda BV, Chu L, Curtin JM, et al. Myalgic Encephalomyelitis/Chronic Fatigue Syndrome: Essentials of Diagnosis and Management. Mayo Clin Proc. 2021;96(11):2861–78. doi: 10.1016/j.mayocp.2021.07.004 34454716

[9] Chu L, Valencia IJ, Garvert DW, Montoya JG. Onset Patterns and Course of Myalgic Encephalomyelitis/Chronic Fatigue Syndrome. Front Pediatr. 2019;7:12. doi: 10.3389/fped.2019.00012 30805319 PMC6370741

[10] Cairns R, Hotopf M. A systematic review describing the prognosis of chronic fatigue syndrome. Occup Med (Lond). 2005;55(1):20–31. doi: 10.1093/occmed/kqi013 15699087

[11] Bartlett C, Hughes JL, Miller L. Living with myalgic encephalomyelitis/chronic fatigue syndrome: Experiences of occupational disruption for adults in Australia. Br J Occup Ther. 2022;85(4):241–50. doi: 10.1177/03080226211020656 40337214 PMC12033770

[12] Balinas C, Eaton-Fitch N, Maksoud R, Staines D, Marshall-Gradisnik S. Impact of Life Stressors on Myalgic Encephalomyelitis/Chronic Fatigue Syndrome Symptoms: An Australian Longitudinal Study. Int J Environ Res Public Health. 2021;18(20):10614. doi: 10.3390/ijerph182010614 34682360 PMC8535742

[13] Castro-Marrero J, Faro M, Zaragozá MC, Aliste L, De Sevilla TF, Alegre J. Unemployment and work disability in individuals with chronic fatigue syndrome/myalgic encephalomyelitis: a community-based cross-sectional study from Spain. BMC Public Health. 2019;19(1):840. doi: 10.1186/s12889-019-7225-z 31253111 PMC6599355

[14] Weigel B, Eaton-Fitch N, Passmore R, Cabanas H, Staines D, Marshall-Gradisnik S. Gastrointestinal symptoms, dietary habits, and the effect on health-related quality of life among Australian Myalgic Encephalomyelitis/Chronic Fatigue Syndrome patients: A cross-sectional study. Ann Epidemiol Public Health. 2020;3(1):1045.

[15] Eaton-Fitch N, Johnston SC, Zalewski P, Staines D, Marshall-Gradisnik S. Health-related quality of life in patients with myalgic encephalomyelitis/chronic fatigue syndrome: an Australian cross-sectional study. Qual Life Res. 2020;29(6):1521–31. doi: 10.1007/s11136-019-02411-6 31970624 PMC7253372

[16] Johnston SC, Staines DR, Marshall-Gradisnik SM. Epidemiological characteristics of chronic fatigue syndrome/myalgic encephalomyelitis in Australian patients. Clin Epidemiol. 2016;8:97–107. doi: 10.2147/CLEP.S96797 27279748 PMC4878662

[17] Kingdon CC, Bowman EW, Curran H, Nacul L, Lacerda EM. Functional Status and Well-Being in People with Myalgic Encephalomyelitis/Chronic Fatigue Syndrome Compared with People with Multiple Sclerosis and Healthy Controls. Pharmacoecon Open. 2018;2(4):381–92. doi: 10.1007/s41669-018-0071-6 29536371 PMC6249197

[18] Zhao T, Cox IA, Ahmad H, Campbell JA, Hensher M, Palmer AJ, et al. The economic burden of myalgic encephalomyelitis/chronic fatigue syndrome in Australia. Aust Health Rev. 2023;47(6):707–15. doi: 10.1071/AH23106 38011828

[19] Close S, Marshall-Gradisnik S, Byrnes J, Smith P, Nghiem S, Staines D. The Economic Impacts of Myalgic Encephalomyelitis/Chronic Fatigue Syndrome in an Australian Cohort. Front Public Health. 2020;8:420. doi: 10.3389/fpubh.2020.00420 32974259 PMC7472917

[20] Reilly A, Buchanan R. ME/CFS National Disability Agreement Review Submission. 2018.

[21] Melby L, das Nair R. “We have no services for you… so you have to make the best out of it”: A qualitative study of Myalgic Encephalomyelitis/Chronic Fatigue Syndrome patients’ dissatisfaction with healthcare services. Health Expect. 2023;27(1):e13900. doi: 10.1111/hex.13900 37905602 PMC10726260

[22] De Carvalho Leite JC, De L Drachler M, Killett A, Kale S, Nacul L, McArthur M, et al. Social support needs for equity in health and social care: a thematic analysis of experiences of people with chronic fatigue syndrome/myalgic encephalomyelitis. Int J Equity Health. 2011;10(1). doi: 10.1186/1475-9276-10-46 22044797 PMC3229491

[23] Sommerfelt K, Schei T, Angelsen A. Severe and Very Severe Myalgic Encephalopathy/Chronic Fatigue Syndrome ME/CFS in Norway: Symptom Burden and Access to Care. J Clin Med. 2023;12(4):1487. doi: 10.3390/jcm12041487 36836022 PMC9963221

[24] Weigel B, Eaton-Fitch N, Thapaliya K, Marshall-Gradisnik S. Illness presentation and quality of life in myalgic encephalomyelitis/chronic fatigue syndrome and post COVID-19 condition: a pilot Australian cross-sectional study. Qual Life Res. 2024;33(9):2489–507. doi: 10.1007/s11136-024-03710-3 38961009 PMC11390810

[25] Johnston SC, Brenu EW, Hardcastle SL, Huth TK, Staines DR, Marshall-Gradisnik SM. A comparison of health status in patients meeting alternative definitions for chronic fatigue syndrome/myalgic encephalomyelitis. Health Qual Life Outcomes. 2014;12:64. doi: 10.1186/1477-7525-12-64 24886213 PMC4008489

[26] Australian Public Service Commission. Definition of disability. Canberra, Australia: Commonwealth of Australia. 2019. https://www.apsc.gov.au/working-aps/diversity-and-inclusion/disability/definition-disability#:~:text=Persons%20are%20considered%20to%20have,and%20restricts%20everyday%20activities3

[27] Weigel B, Eaton-Fitch N, Passmore R, Cabanas H, Staines D, Marshall-Gradisnik S. A preliminary investigation of nutritional intake and supplement use in Australians with myalgic encephalomyelitis/chronic fatigue syndrome and the implications on health-related quality of life. Food Nutr Res. 2021;65:10.29219/fnr.v65.5730. doi: 10.29219/fnr.v65.5730 34262415 PMC8254462

[28] Weigel B, Eaton-Fitch N, Thapaliya K, Marshall-Gradisnik S. A pilot cross-sectional investigation of symptom clusters and associations with patient-reported outcomes in Myalgic Encephalomyelitis/Chronic Fatigue Syndrome and Post COVID-19 Condition. Qual Life Res. 2024;33(12):3229–43. doi: 10.1007/s11136-024-03794-x 39361124 PMC11599292

[29] Griffith University. Griffith University Research Ethics Manual. 2018. https://www.griffith.edu.au/research/research-services/research-ethics-integrity/human/gurem

[30] National Health and Medical Research Council, Australian Research Council, Universities Australia. National Statement on Ethical Conduct in Human Research. Canberra, Australia: National Health and Medical Research Council. 2023. https://www.nhmrc.gov.au/about-us/publications/national-statement-ethical-conduct-human-research-2023

[31] World Medical Association. World Medical Association Declaration of Helsinki: Ethical principles for medical research involving human subjects. JAMA. 2013;310(20):2191–4. doi: 10.1001/jama.2013.281053 24141714

[32] Von Elm E, Altman DG, Egger M, Pocock SJ, Gøtzsche PC, Vandenbroucke JP. The Strengthening the Reporting of Observational Studies in Epidemiology (STROBE) statement: guidelines for reporting observational studies. J Clin Epidemiol. 2008;61(4):344–9. doi: 10.1016/j.jclinepi.2007.11.008 18313558

[33] Abernethy AP, Shelby-James T, Fazekas BS, Woods D, Currow DC. The Australia-modified Karnofsky Performance Status (AKPS) scale: a revised scale for contemporary palliative care clinical practice. BMC Palliat Care. 2005;4(1). doi: 10.1186/1472-684x-4-7 16283937 PMC1308820

[34] Bell DS. The doctor’s guide to chronic fatigue syndrome: Understanding, treating, and living with CFIDS. 1995.

[35] Paralyzed Veterans of America. Fatigue and multiple sclerosis: Evidence-based management strategies for fatigue in multiple sclerosis. Washington, United States: Paralyzed Veterans of America. 1998.

[36] Üstün TB, Kostanjsek N, Chatterji S, Rehm J, World Health Organization. Measuring health and disability: Manual for WHO Disability Assessment Schedule (WHODAS 2.0). Geneva, Switzerland: World Health Organization. 2010. https://www.who.int/publications/i/item/measuring-health-and-disability-manual-for-who-disability-assessment-schedule-(-whodas-2.0)

[37] Wagner D, Nisenbaum R, Heim C, Jones JF, Unger ER, Reeves WC. Psychometric properties of the CDC Symptom Inventory for assessment of Chronic Fatigue Syndrome. Popul Health Metrics. 2005;3(1). doi: 10.1186/1478-7954-3-8 16042777 PMC1183246

[38] Ware JE Jr. SF-36 health survey update. Spine (Phila Pa 1976). 2000;25(24):3130–9. doi: 10.1097/00007632-200012150-00008 11124729

[39] Wolfe F, Clauw DJ, Fitzcharles M-A, Goldenberg DL, Katz RS, Mease P, et al. The American College of Rheumatology preliminary diagnostic criteria for fibromyalgia and measurement of symptom severity. Arthritis Care Res (Hoboken). 2010;62(5):600–10. doi: 10.1002/acr.20140 20461783

[40] Harris PA, Taylor R, Minor BL, Elliott V, Fernandez M, O’Neal L, et al. The REDCap consortium: Building an international community of software platform partners. J Biomed Inform. 2019;95:103208. doi: 10.1016/j.jbi.2019.103208 31078660 PMC7254481

[41] Harris PA, Taylor R, Thielke R, Payne J, Gonzalez N, Conde JG. Research electronic data capture (REDCap)--a metadata-driven methodology and workflow process for providing translational research informatics support. J Biomed Inform. 2009;42(2):377–81. doi: 10.1016/j.jbi.2008.08.010 18929686 PMC2700030

[42] IBM Corp. IBM Statistical Package for the Social Sciences, Version 29.0. 2022.

[43] Lang TA, Altman DG. Basic statistical recording for articles published in biomedical journals: “The Statistical Analyses and Methods in the Published Literature” or “The SAMPL Guidelines.”. In: Smart P, Maisonneuve H, Polderman A. Science Editor’s Handbook. 2nd ed. Cornwall, United Kingdom: European Association of Science Editors. 2013.

[44] Ranganathan P. An Introduction to Statistics: Choosing the Correct Statistical Test. Indian J Crit Care Med. 2021;25(Suppl 2):S184–6. doi: 10.5005/jp-journals-10071-23815 34345136 PMC8327789

[45] Tastle WJ, Wierman MJ, Dumdum UR. Ranking ordinal scales using the consensus measure. IIS. 2005;6(2):96–102. doi: 10.48009/2_iis_2005_96-102

[46] Stevenson C. SF-36: Interim norms for Australian data. Canberra, Australia: Australian Institute of Health and Welfare. 1996. https://www.aihw.gov.au/reports/corporate-publications/sf-36-interim-norms-for-australian-data/contents/table-of-contents

[47] Stensen K, Lydersen S. Internal consistency: from alpha to omega? Tidsskr Nor Laegeforen. 2022;142(12):10.4045/tidsskr.22.0112. doi: 10.4045/tidsskr.22.0112 36066232

[48] Weigel B, Inderyas M, Eaton-Fitch N, Thapaliya K, Marshall-Gradisnik SM. Health-related quality of life in Myalgic Encephalomyelitis/Chronic Fatigue Syndrome and Post COVID-19 Condition: A systematic review. J Transl Med. 2025;23:318. doi: 10.1186/s12967-025-06131-z 40075382 PMC11905571

[49] Jo Delaney L. Patient-centred care as an approach to improving health care in Australia. Collegian. 2018;25(1):119–23. doi: 10.1016/j.colegn.2017.02.005

[50] Wiles LK, Kay D, Luker JA, Worley A, Austin J, Ball A, et al. Consumer engagement in health care policy, research and services: A systematic review and meta-analysis of methods and effects. PLoS One. 2022;17(1):e0261808. doi: 10.1371/journal.pone.0261808 35085276 PMC8794088

[51] Department of Health and Aged Care. National Consumer Engagement Strategy for Health and Wellbeing. Canberra, Australia: Commonwealth of Australia. 2023. https://www.health.gov.au/resources/publications/national-consumer-engagement-strategy-for-health-and-wellbeing?language=en

[52] World Health Organization. Framework on integrated, people-centred health services — report by the secretariat. Geneva, Switzerland: World Health Organization. 2016. https://www.who.int/news/item/28-05-2016-world-health-assembly-adopts-framework-on-integrated-people-centred-health-services

[53] Biddle N, Gray M, Rehill P. Mental health and wellbeing during the COVID-19 period in Australia. Canberra, Australia: Australian National University Centre for Social Research and Methods. 2022. https://polis.cass.anu.edu.au/research/publications/mental-health-and-wellbeing-during-covid-19-period-australia

[54] Mercieca-Bebber R, Campbell R, Fullerton DJ, Kleitman S, Costa DSJ, Candelaria D, et al. Health-related quality of life of Australians during the 2020 COVID-19 pandemic: a comparison with pre-pandemic data and factors associated with poor outcomes. Qual Life Res. 2022;32(2):339–55. doi: 10.1007/s11136-022-03222-y 35989367 PMC9393100

[55] Jason L, Brown M, Evans M, Anderson V, Lerch A, Brown A, et al. Measuring substantial reductions in functioning in patients with chronic fatigue syndrome. Disabil Rehabil. 2011;33(7):589–98. doi: 10.3109/09638288.2010.503256 20617920 PMC3170036

[56] Mather H, Guo P, Firth A, Davies JM, Sykes N, Landon A, et al. Phase of Illness in palliative care: Cross-sectional analysis of clinical data from community, hospital and hospice patients. Palliat Med. 2018;32(2):404–12. doi: 10.1177/0269216317727157 28812945 PMC5788082

[57] Stoothoff J, Gleason K, McManimen S, Thorpe T, Jason LA. Subtyping Patients with Myalgic Encephalomyelitis (ME) and Chronic Fatigue Syndrome (CFS) By Course of Illness. J Biosens Biomark Diagn. 2017;2(1):10.15226/2575-6303/2/1/00113. doi: 10.15226/2575-6303/2/1/00113 29204592 PMC5710812

[58] Stussman B, Williams A, Snow J, Gavin A, Scott R, Nath A, et al. Characterization of Post–exertional Malaise in Patients With Myalgic Encephalomyelitis/Chronic Fatigue Syndrome. Front Neurol. 2020;11. doi: 10.3389/fneur.2020.01025 33071931 PMC7530890

[59] Sasso EM, Muraki K, Eaton-Fitch N, Smith P, Lesslar OL, Deed G, et al. Transient receptor potential melastatin 3 dysfunction in post COVID-19 condition and myalgic encephalomyelitis/chronic fatigue syndrome patients. Mol Med. 2022;28(1). doi: 10.1186/s10020-022-00528-y 35986236 PMC9388968

[60] Marshall-Gradisnik S, Sasso EM, Eaton-Fitch N, Smith P, Baraniuk JN, Muraki K. Novel characterization of endogenous transient receptor potential melastatin 3 ion channels from Gulf War Illness participants. PLoS One. 2024;19(6):e0305704. doi: 10.1371/journal.pone.0305704 38917121 PMC11198784

[61] Castro-Marrero J, Faro M, Aliste L, Sáez-Francàs N, Calvo N, Martínez-Martínez A, et al. Comorbidity in Chronic Fatigue Syndrome/Myalgic Encephalomyelitis: A Nationwide Population-Based Cohort Study. Psychosomatics. 2017;58(5):533–43. doi: 10.1016/j.psym.2017.04.010 28596045

[62] Pheby DFH, Saffron L. Risk factors for severe ME/CFS. Biol Med. 2009;1(4):50–74. https://bnu.repository.guildhe.ac.uk/id/eprint/9823

[63] Vaes AW, Van Herck M, Deng Q, Delbressine JM, Jason LA, Spruit MA. Symptom-based clusters in people with ME/CFS: an illustration of clinical variety in a cross-sectional cohort. J Transl Med. 2023;21(1):112. doi: 10.1186/s12967-023-03946-6 36765375 PMC9921324

[64] Komaroff AL, Lipkin WI. Insights from myalgic encephalomyelitis/chronic fatigue syndrome may help unravel the pathogenesis of postacute COVID-19 syndrome. Trends Mol Med. 2021;27(9):895–906. doi: 10.1016/j.molmed.2021.06.002 34175230 PMC8180841

[65] Davis HE, McCorkell L, Vogel JM, Topol EJ. Long COVID: major findings, mechanisms and recommendations. Nat Rev Microbiol. 2023;21(3):133–46. doi: 10.1038/s41579-022-00846-2 36639608 PMC9839201

