# Supplementary material for: Sustained illness burden over time among Australians with myalgic encephalomyelitis/chronic fatigue syndrome
Source: PLoS One. 2025 Dec 29;20(12):e0338433. doi: 10.1371/journal.pone.0338433 (PMC12747376; doi:10.1371/journal.pone.0338433)
Supplement: S1 Text — (DOCX) [file pone.0338433.s005.docx]

## Comparisons between participants and non-participants

The sociodemographic and illness characteristics of the n=32 pwME/CFS who participated in the present study were compared with those of the non-participants who consented to the future use of their data to identify potential volunteer biases. Data were available for n=19 participants lost to follow-up and n=18 ineligible respondents. The categorical variables compared between the three cohorts (pwME/CFS, participants lost to follow-up and ineligible respondents) included the presence of comorbid entities, sex at birth, ethnicity, country of birth, education status, current employment status and access to social support. These categorical variables were compared between the participants and non-participants with omnibus statistical tests of independence, including Chi-square and Fisher-Freeman-Halton tests. Where these omnibus tests returned statistical significance, pairwise post-hoc Chi-square and Fisher’s exact tests were performed for all possible pairings of the three cohorts with all possible pairings of the dependent variable.

Nonparametric continuous variables analysed across the three cohorts included BMI, illness duration and hours of domestic work per week. These were compared between the participants and non-participants with the omnibus Kruskal-Wallis *H* test. For Kruskal-Wallis *H* tests returning statistical significance, post-hoc Dunn-Bonferroni tests were performed and adjusted for multiple comparisons using the Benjamini-Hochberg correction to identify the cohorts between which significant differences lay. Parametric continuous variables (including age and hours of paid work per week) were compared between the three cohorts with independent samples one-way ANOVA tests. Post-hoc Dunn-Bonferroni tests were performed for significant ANOVA models and adjusted for multiple comparisons using the Benjamini-Hochberg correction.

Few sociodemographic and illness characteristics differed significantly between the n=32 pwME/CFS who participated in the present study with the n=19 participants lost to follow-up and n=18 ineligible respondents. Significant differences were observed among the three cohorts for illness duration and hours of paid work per week. The significantly shorter illness duration (p=0.011, unadjusted) among the ineligible respondents when compared with both the pwME/CFS and the participants lost to follow-up is unsurprising, as n=11/18 (61.1%) of this cohort reported the onset of ME/CFS-like symptoms after acute Coronavirus Disease 2019 (COVID-19) illness. As the relationship between ME/CFS and Long COVID remains incompletely defined [1, 64, 65], these n=11 participants were excluded from the present study to ensure the generalisability of the results observed to pwME/CFS. Excluding the ineligible respondents with Long COVID, the remaining n=7/18 (38.9%) ineligible respondents had a comparable median illness duration of 16.67 (Q1–Q3=4.42–32.42) years when compared with the pwME/CFS and participants lost to follow-up (p=0.86, unadjusted).

The participants lost to follow-up but not the ineligible respondents spent significantly more hours in paid work per week than the pwME/CFS (p=0.039, unadjusted). Whilst over one-quarter and half of the employed participants lost to follow-up (n=3/8, 27.5%) and ineligible respondents (n=5/10, 50.0%), respectively, reported working full-time (p=0.016, unadjusted), employment status was not significantly different when compared with the pwME/CFS after adjusting for multiple comparisons. PwME/CFS reported a significantly higher median number of comorbid entities (M=5, Q1–Q3=3–7) when compared with the participants lost to follow-up (M=2, Q1–Q3=1–3, p<0.05) and ineligible respondents (M=3, Q1–Q3=2–5, p<0.05). Prevalence was significantly higher for FM and asthma (both p<0.001) and lower for depression (p=0.013) among the participants when compared with the non-participant cohorts. However, the non-participants’ medical history was only collected during screening for the ineligible respondents and at baseline for the participants lost to follow-up. Those who completed all three questionnaires continued to update their medical history throughout the study, which may explain the increased reporting of comorbid entities.
